# Osteoclast-derived apoptotic bodies show extended biological effects of parental cell in promoting bone defect healing

**DOI:** 10.7150/thno.45170

**Published:** 2020-05-22

**Authors:** Qinyu Ma, Mengmeng Liang, Nathachit Limjunyawong, Yang Dan, Junchao Xing, Jianmei Li, Jianzhong Xu, Ce Dou

**Affiliations:** 1 Department of Orthopedics, Southwest Hospital, Third Military Medical University, Chongqing 400038, China.; 2 Department of Biomedical Materials Science, Third Military Medical University, Chongqing 400038, China.; 3 The Solomon H. Snyder Department of Neuroscience, Johns Hopkins University School of Medicine, Baltimore, Maryland 21205, USA.; 4 Department of Orthopedics, Union Hospital, Tongji Medical College, Huazhong University of Science and Technology, Wuhan 430022, China.; 5 Department of Orthopedic Surgery, Johns Hopkins University School of Medicine, Baltimore, Maryland 21205, USA.

**Keywords:** Apoptotic body, Osteoclast, Vesicle bioinformatics, Cell coupling, Bone remodeling

## Abstract

Apoptotic bodies (ABs) traditionally considered as garbage bags that enclose residual components of dead cells are gaining increasing attentions due to their potential roles in intercellular communications. In bone turn over, at the end of bone resorption phase, most osteoclasts undergo apoptosis, generating large amounts of ABs. However, it remains unclear of the role of osteoclast-derived ABs in bone remodeling.

**Methods**: Staurosporine (STS) was used to apoptotic induction and differential centrifugation was used to isolate ABs. Western blotting, flowcytometry and Transmission electron microscopy (TEM) were performed for ABs identification, while whole transcriptome of ABs from osteoclasts at different stages was detected by RNA-seq. VENN analysis and gene set enrichment analysis (GSEA) were performed to compare the profile similarities between ABs and parental cells. *In vitro* efficacy of ABs on angiogenesis and osteogenesis were evaluated by tube formation assay and ALP staining. *In vivo*, calvarial defect mice model was used to assess the effects of ABs-modified decalcified bone matrix (DBM) scaffolds on angiogenesis and osteogenesis.

**Results**: Here we mapped the whole transcriptome paralleled with small RNA profiling of osteoclast derived ABs at distinct differentiation stages. Whole transcriptome analysis revealed significant differences in RNA signatures among the ABs generated from osteoclasts at different stages. By comparing with parental osteoclast RNA profiles, we found that the transcriptome of ABs exhibited high similarities with the corresponding parental cells. Functionally, *in vitro* and *in vivo* studies showed that similar with the parental cells, pOC-ABs potentiated endothelial progenitor cell proliferation and differentiation, whereas mOC-ABs promoted osteogenic differentiation. The inherited biological effects of ABs were shown mediated by several enriched lncRNAs of which the interference abolished AB functions.

**Conclusions**: Our study revealed the total RNA profiles of osteoclast derived ABs and demonstrated their biological functions. Both gene set and functional analysis indicated that osteoclast derived ABs are biologically similar with the parental cells suggesting their bridging role in osteoclast-osteoblast coupling in bone remodeling.

## Introduction

Extracellular vesicles (EVs) play a vital role in intercellular communication and signal transduction by transferring bioactive molecules from parental cells to recipient cells 1-3. EVs are widely classified into three subtypes: exosomes, microvesicles (MVs) and apoptotic bodies (ABs). The generation of all three EV subtypes is associated with cytoskeletal reorganization and membrane budding 4,5. Nevertheless, there are profound differences among these subtypes including their size, content and biogenesis. Exosomes are generally between 30 to 150 nm in diameter with specific lipid bilayer membrane structure, MVs are larger between 100 to 1000 nm in diameter and ABs are the largest between 1 to 5 μm in diameter 6,7. Exosomes and MVs are mostly produced by live cells, however; ABs are generated from apoptotic cells. Recent studies have demonstrated that various RNA species (including mRNAs, miRNAs, and lncRNAs) entrapped within EVs can be transferred from donor to acceptor cells and interfere in gene expression in the latter 8,9. While most of the above studies focused on exosomes and MVs, few studies reported the RNA function in ABs.

Cell apoptosis is a process of programmed cell death characterized by significant morphological changes and is considered to play a vital role in cell turnover, normal development and function of immune system 10,11. During apoptosis, the nucleus and cytoplasm of dying cell condense and segment under the contraction of actin skeleton and eventually generate ABs 5,10. Generally, packaging of intracellular organelles like endoplasmic reticulum, mitochondria and nuclear contents into ABs during apoptosis is considered to be a random process 12,13. The contents of ABs are stochastic and variable, and may contain nucleic acids, proteins and lipids 14-16. Some autoantigens can also be transferred into ABs during cell apoptosis such as histone 3, histone 2B, complement C1q C Chain (C1QC) and complement component 3B (C3B), thereby become specific markers of ABs 17,18. Increasing evidences suggested that ABs have regulatory roles in inflammation, autoimmune and cancer 18-20, raising the possibility that ABs may be key factors of intercellular communications. However, how ABs exert their biological functions remains unclear and studies are lack in revealing the contents in detail within ABs.

Osteoclast is the only known bone resorbing cell regulating bone remodeling and homeostasis. Stimulated by receptor activator of nuclear factor κB ligand (RANKL) and macrophage-colony stimulating factor (M-CSF), monocytes/macrophage progenitor cells first differentiate to mononuclear preosteoclasts (pOCs, 24 h after stimulation), upon continuous induction, pOC fuses with each other and differentiate to multinucleated mature osteoclasts (mOCs, 96 h after stimulation) 21. Although the nomenclature changes little, the function of pOC and mOC is completely different. Giant mOC is terminally differentiated and highly efficient in bone resorption, while pOC at its transition stage barely resorbs bone and secretes cytokines for anabolism 22. The most well recognized regulatory role of pOC is the pro-angiogenesis activity via secreting cytokines including PDGF-BB 23. As for mOC, the pro-osteogenic activities via RANKL reverse signaling couples bone resorption and bone formation 24, 25. Our group also previously characterized that pOC and mOC have significantly different transcriptome and small RNA signatures 26. The life span of osteoclasts is short relative to other bone cells of about two weeks 27,28. In bone turn over, at the end of bone resorption phase, most osteoclasts undergo apoptosis, generating a large amount of ABs and marking the termination of bone resorption and the initiation of bone formation 29. In this regard, osteoclast derived ABs periodically occurred in the bone remodeling microenvironment in large scale. However, whether these osteoclast-derived ABs participate in the process of bone remodeling and their potential biological function is completely unknown.

In this study, we mapped the expression of the whole-transcriptome paralleled with small RNA profiles in osteoclast derived ABs. For the first time, we demonstrated the presence of non-coding RNAs in ABs suggesting their diverse regulatory role in intercellular communication. By comparing with the total RNA profiles with the corresponding parental cells, a significant signature similarity was revealed. This similarity was further confirmed functionally by *in vitro* and *in vivo* tests.

## Materials and methods

### Osteoclast differentiation assay

For TRAP staining, bone marrow macrophage (BMM) were incubated in 96-well plates at a density of 5×10^3^ cells per well with M-CSF (50 ng/mL) and RANKL (100 ng/mL). At 0 h, 24 h and 96 h after stimulation, cells were fixed in 4% paraformaldehyde for 20 min and then stained with TRAP staining solution (0.1 mg/ml of naphthol phosphate disodium salt, 0.3 mg/mL of Fast Red Violet zinc chloride stain) according to the manufacturers' instructions. Relative TRAP activity was analyzed by colorimetry. For immunofluorescent (IF) staining, BMM were incubated in 96-well plates at a density of 5×10^3^ cells per well with M-CSF (50 ng/mL) and RANKL (100 ng/mL) for osteoclastogenesis. Specific procedures have been described in previous study 25,30. In short, cells were washed, fixed and permeabilized with 0.2% Triton X-100. After blocking, the cells were incubated with antibody against vinculin (1:500 diluted in blocking solution) for an hour at 37 °C. Then, nuclei counterstaining was conducted by staining with DAPI (1:1000) for 10 minutes followed by fluorescence microscopy and confocal microscopy observation.

### ABs generation and isolation

Staurosporine (obtained from MCE, Med Chem Express, diluted to 0.5μM) was added to induce cell apoptosis for 3 hours at 37 °C. After apoptosis induction, sequential centrifugation and sequential filtration was conducted to separate ABs. Specifically, media was collected from petri dish and centrifuged at 300×g for 10 min to eliminate cell debris. After that, the remaining supernatant was centrifuged at 3000×g for 30 min to pellet the AB-sized extracellular vesicles. After AB-sized extracellular vesicles from osteoclasts and STS-treated osteoclasts were separated, AB-sized extracellular vesicles were labelled with Annexin V-FITC in 500 μL binding buffer for 30 min at 21 °C. Next, AB-sized extracellular vesicles were centrifuged at 3000×g for 30 min in order to pellet again to remove binding buffer for the further identification and calculation.

### ABs identification and evaluation

AnnexinV-FITC and PI (included in apoptosis assay kit, purchased from Sigma) were used to mark ABs. The exposure of phosphatidylserine to the vesicle surface caused by apoptosis was measured by AnnexinV-FITC (20 mg/mL), and nuclear granularity and hypochromicity were examined by PI (50 mg/mL). Flow cytometry was then used to analyze and quantify the purity of separated ABs by event number quantification of AB-size vesicles. After quantification, AB-media was generated by adding 1×10^5^ ABs into 1mL of complete medium for further *in vitro* study.

### Total RNA sequencing

Total AB RNA was isolated using RNeasy mini kit (Qiagen, Germany). Paired-end libraries were synthesized by using the TruSeq™ RNA Sample Preparation Kit (Illumina, USA) following TruSeq™ RNA Sample Preparation Guide. Briefly, the poly-A containing mRNA molecules were purified using poly-T oligo-attached magnetic beads. Following purification, the mRNA was fragmented into small pieces using divalent cations at 94 ℃ for 8 min. The cleaved RNA fragments were converted into first strand cDNA using reverse transcriptase and random primers. This was followed by second strand cDNA synthesis using DNA Polymerase I and RNase H. These cDNA fragments then underwent an end repair process, the addition of a single 'A' base, and then ligation of the adapters. The products were then purified and enriched with PCR to create the final cDNA library. Purified libraries were quantified by Qubit® 2.0 Fluorometer (Life Technologies, USA) and validated by Agilent 2100 bioanalyzer (Agilent Technologies, USA) to confirm the insert size and calculate the mole concentration. Cluster was generated by cBot with the library diluted to 10 pM and then was sequenced on the Illumina NovaSeq 6000 (Illumina, USA). All raw data of RNA-seq were uploaded to GEO database with accession number of GSE132230.

### Bioinformatics analysis and visualization

Data processing and statistical testing was conducted with the R programming language. All differentially expressed RNAs were screened with FDR adjusted p-value less than 0.05 and fold change more than 2.0. All input data were well prepared and transformed to readable formats before graphic drawing using R. The standardized relative expression ratios were calculated followed by a log2 transformation for further normalization between three samples. Hierarchical clustering heatmaps were performed using the complete agglomeration method of h-clust as implemented in the heatmap2 R package. Scatter plots were drawn by ggplot2 R package and VENN analysis was conducted by VENN diagram R package, respectively. Results of Gene ontology (GO) and KEGG pathway enrichment analysis were generated using clusterProfiler R package followed by preparation and processing of input data, eventually visualized using ggplot2 R package. Gene set enrichment (GSEA) analysis 31 was performed by clusterProfiler R package using our publicly available data (accession number GSE72478) 26 and predefined gene sets of GO biological process.

### Tube formation assay

To prepare Matrigel-coated plate, 24-well plate was placed on ice for 20-30 min and 250 µL of Matrigel (BD Biosciences, San Jose, CA, USA) was added into each well of the plate and incubated at 37 °C and 5% CO_2_ for 30 min to solidify Matrigel. After Matrigel polymerization, 1×10^4^ endothelial cells were seeded on the Matrigel with 500 μL AB-medium, and cells cultured with α-MEM as control. After incubation at 37 °C for 2 and 6 hours, tube network was observed in 5 randomly selected microscopic fields using an inverted microscope attached to a digital camera (Leica DM1600, Wetzlar, Germany) and quantified by measuring the cumulative tube lengths by ImageJ software.

### Fabrication of AB-modified DBM scaffolds

Decalcified bone matrix (DBM) were prepared from bovine limbs as previously reported 32. The DBM were cut into 2.5 mm cubes to fit the calvarial defect size. After being immersed in 75% ethanol for 2 h, the DBM were washed three times with PBS and then coated with 10 μg/mL fibronectin (Sigma) overnight at 37 °C. As for incubation, 50 μg of isolated ABs were resuspended in 50 μL of control media, subsequently followed by the incubation of DBM with AB-contained media for 6 hours at 37 °C. The DBM were then air dried and stored at 80 °C until they were used in experiments.

### Mice

Mouse experimental protocols were performed with the approval of the Third Military Medical University (TMMU) Animal Ethics Experimentation Committee. Male C57BL/6 mice (11-week-old) were obtained from the Animal Institution of TMMU. After euthanasia, bone marrow cells were isolated and cultured with M-CSF (50 ng/ml) for 48 h to obtain BMM precursor cells. The endothelial progenitor cells (EPCs) were separated from the bone marrow mononuclear cells (BMPCs) of C57BL/6 mice as previously described 33. After isolation, cells were cultured in α-minimal essential medium (α-MEM) (Gibco, BRL, Gaithersburg USA) containing 10% fetal bovine serum (FBS) and 1% Penicillin-streptomycin solution (Gibco, BRL, Gaithersburg USA) at 37 °C in a 5% CO2 humidified incubator. As for cranial drilling, mice were anesthetized by intraperitoneal injection of chloral hydrate (Sigma Aldtich, 2mg/100g), a sagittal median incision of 1.5 cm was made and two 2.5 mm-sized defects were created symmetrically on both sides of the calvaria bone using a dental micro-drill. Afterwards, the DBM pre-incubated with different ABs for 6h was implanted into the bone defect area. Mice were randomly divided into four groups: DBM repair (Vehicle, n = 8), BMM-AB-DBM repair (BMM-ABs, n = 8), pOC-AB-DBM repair (pOC-ABs, n = 8), mOC-AB-DBM repair (pOC-ABs, n = 8). After 2 weeks (for angiogenesis evaluation) or 4 weeks (for osteogenesis evaluation) of normal feeding, mice were euthanized for further evaluation. The calvarial bone of mice were removed and waited for further detection.

### Micro-CT

Image of the whole calvarial bone was obtained using a Bruker MicroCT Skyscan 1272 system (Kontich, Belgium) with an isotropic voxel size of 10.0 μm. Scanning was performed in 4% paraformaldehyde using a 60 kV X-ray tube with an X-ray intensity of 166 μA and an exposure time of 1700 ms. μCT scans of the whole calvarial bone of mice were performed using isotropic voxel sizes of 148μm. A 3D reconstruction of the images was conducted for the region of interest containing the DBM. Reconstruction was performed using a Nrecon (Kontich, Belgium). 3D images were acquired from contoured 2D images by methods based on distance transformation of the gray scale original images (Ver. 3.0.0, CTvox, Kontich, Belgium). 3D and 2D analysis were performed using software CT Analyser (Ver. 1.15.4.0, Kontich, Belgium). All images presented are representative of the respective groups.

### Statistical analysis

All data are representative of at least three experiments of similar results performed in triplicate unless otherwise indicated. Data are expressed as mean ± SD. One-way ANOVA followed by Student-Newman-Keuls post hoc tests was used to determine the significance of difference between results, with *p < 0.05, **p <0.01 being regarded as significant.

## Results

### Generation and characterization of BMM-ABs, pOC-ABs and mOC-ABs

**Figure 1:**
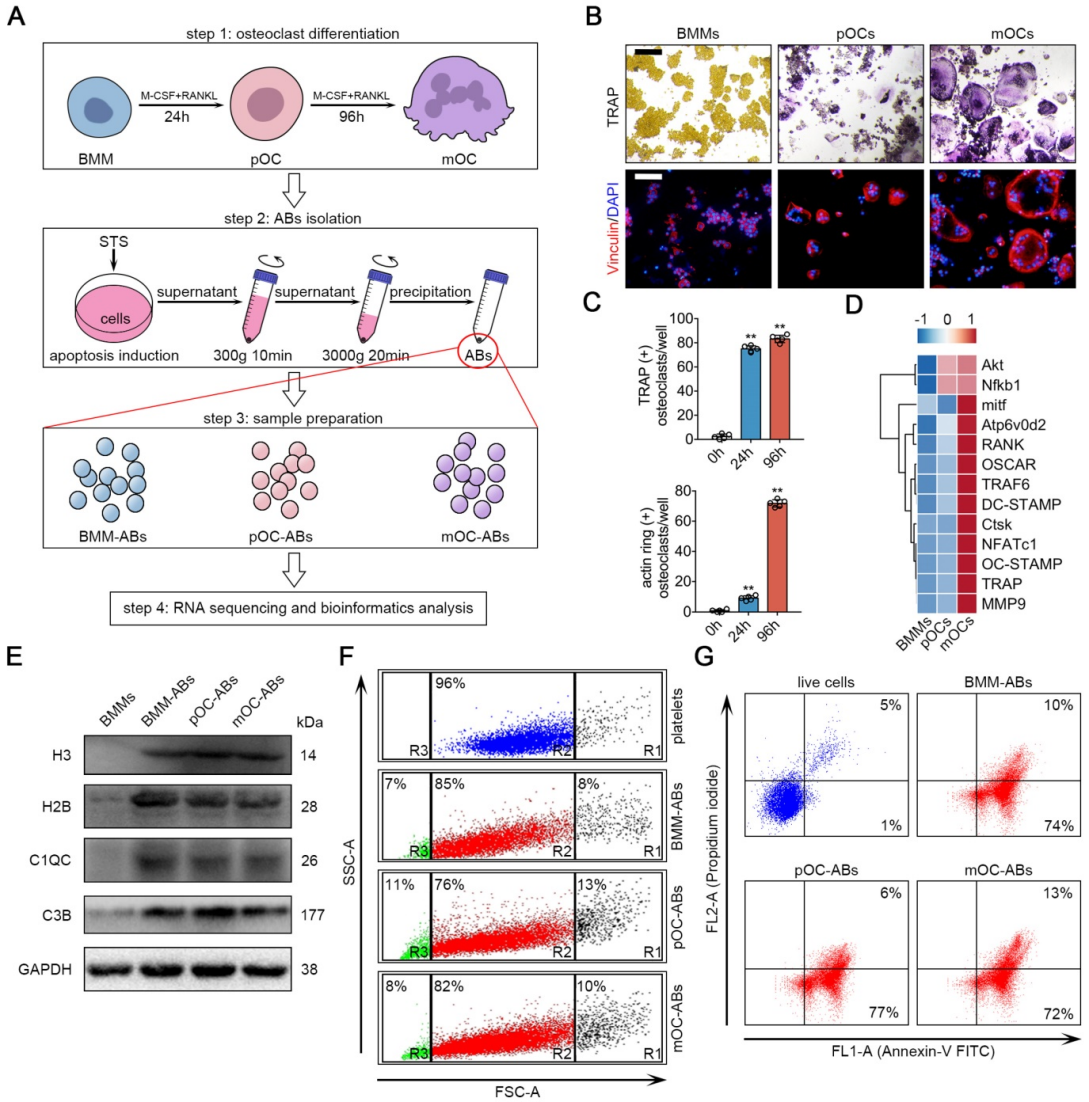
** Generation and characterization of BMM-ABs, pOC-ABs and mOC-ABs.** (**A**) Schematic diagram for osteoclast differentiation and the isolation of ABs from BMMs, pOCs and mOCs. (**B**) Representative TRAP stain and immunofluorescent (IF) staining images of bone marrow macrophages (BMMs), pre-osteoclasts (pOCs) and mature osteoclasts (mOCs). IF stain of vinculin (red) marking actin ring were shown. Bar represents 200 µm. (**C**) Quantification of TRAP positive cells number and actin ring positive cells number per well in B. (**D**) Gene array analysis showed the alteration of osteoclast specific genes expression from BMM to mOC. (**E**) Western blot analysis of H3, H2B, C1QC, C3B and GAPDH in indicated groups. (**F**) FSC/SSC analysis of BMM-ABs, pOC-ABs and mOC-ABs. Platelets (blue) were used as a size marker gating ABs (1-4 µm, gate R2). ABs derived from different periods of osteoclasts contains cell debris (gate R1), ABs (gate R2) and microparticles (gate R3). (**G**) Annexin V/FITC (FL1-A) and PI A (FL2-A) analysis of live cells, BMM-ABs, pOC-ABs and mOC-ABs. The quadrant gates were set on the respective unstained control population. The percentage of events is given in the right corner of indicated region. Images are representative of n = 5 independent experiments. The data in the figures represent the averages ± SD. Significant differences are indicated as * (*p* < 0.05) or ** (*p* < 0.01) paired using Student's t test unless otherwise specified.

We first designed procedures for isolation and characterization of ABs from osteoclasts of different stages (Figure 1A). BMM were isolated and cultured from 11-week old male C57BL/6 mouse hind limbs. For osteoclast differentiation, cells were treated with RANKL and M-CSF to generate pOC (24 h after stimulation) and mOC (96 h after stimulation). TRAP stain and IF stain of actin ring were performed (Figure 1B), and the numbers of TRAP positive cell and actin ring positive cell were quantified to validate the stages of the cells (Figure 1C). The gene array analysis further confirmed that BMM, pOC and mOC have distinct expression profiles of osteoclast specific genes (Figure 1D). To generate AB, we used staurosporine (STS) to induce the apoptosis of BMMs, pOCs and mOCs 34. Light microscopic analyses of viable and apoptotic cells were performed to confirm the apoptotic induction of STS treatment after 3-hr incubation with subcellular fragment count quantification (Figure S1A, S1B). Then, differential centrifugation was conducted to remove large cell debris and other extracellular vesicles 35. ABs-sized EVs were pelleted for characterization and analysis for vesicle size and specific biomarkers. Western blot analysis showed that specific AB markers such as histone 3 (H3), histone 2B (H2B), C1QC and C3B were much more abundant in separated ABs comparing to untreated BMMs (Figure 1E). We then used flow cytometry to analyze the purity and vesicle size of the generated ABs. Platelets whose size (1-4 μm) is similar to ABs were used as a scale for comparison. The results showed that differential centrifugation yielded more than approximately 80% ABs (Figure 1F). We also noticed that about 5%-15% of ABs are PI positive suggesting the containing of DNA fragments. It is worth noting that due to the technology and methodology limitations, AB samples after differential centrifugation still contain a small amount of microparticles (< 1μm, R3 gate in Figure 1F) and cell debris (> 4μm, R1 gate in Figure 1F). Annexin V/PI staining analyzed by flow cytometry revealed that relative to live cell, ABs are mostly (above 80%) positive for Annexin V (Figure 1G, Figure S1C). AB integrity and morphology were then checked using transmission electron microscopy (TEM) (Figure S1D).

### Transcriptional profiling of BMM-ABs, pOC-ABs and mOC-ABs

**Figure 2:**
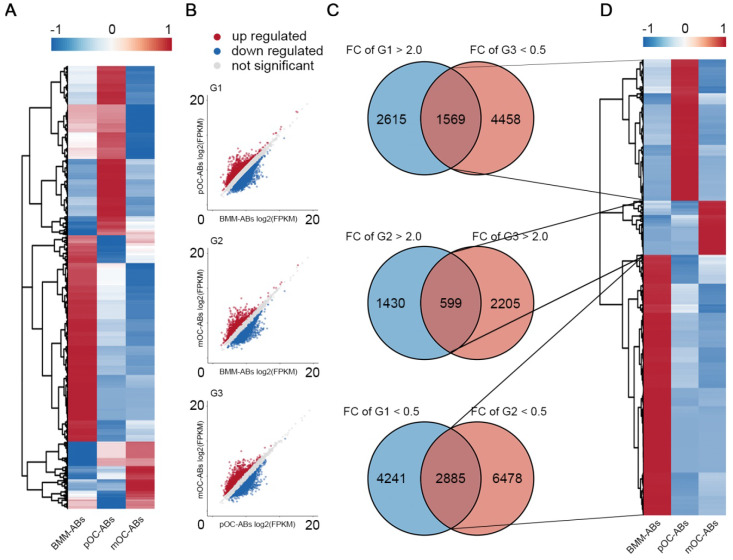
** lncRNA profiling of BMM-ABs, pOC-ABs and mOC-ABs.** (**A**) Hierarchical clustering of all 20,889 lncRNAs expressed in BMM-ABs, pOC-ABs and mOC-ABs. (**B**) Scatter plots of all lncRNAs expressed in three comparison groups were shown. (**C**) VENN analysis screening out lncRNA signatures contained in three ABs. Corresponding lncRNA signatures were screened out using intersection of three comparison groups G1 (pOC-ABs vs BMM-ABs), G2 (mOC-ABs vs BMM-ABs) and G3 (mOC-ABs vs pOC-ABs). FC, fold change. (**D**) Hierarchical clustering of lncRNA signatures in three ABs.

To study the whole transcriptomes of BMM-ABs, pOC-ABs and mOC-ABs, total RNAs of ABs were extracted and RNA-seq was performed using the Illumina NovaSeq 6000. Three comparison groups were established according to the differentiation stages of parental cells, pOC-ABs vs BMM-ABs (G1), mOC-ABs vs BMM-ABs (G2), and mOC-ABs vs pOC-ABs (G3). Based on whether RNAs in the transcriptome can encode proteins, we divided RNAs into protein coding messenger RNAs (mRNAs) and non-protein-coding RNAs (ncRNAs). Differentially expressed RNAs were gated with statistical significance at FDR adjusted *p* < 0.05 and fold change greater than 2.0. For mRNAs, 14,924 transcripts in total were detected and the corresponding expression profiles were presented with clustered heatmap and scatter plots (Figure S2A, S2B). Venn analysis results showed that 1,196, 343 and 1,104 genes were specifically expressed in BMM-ABs, pOC-ABs and mOC-ABs and were hierarchical clustered (Figure S2C, S2D). Top 20 most differentially expressed mRNAs were clustered for better observation (Figure S2E). We then performed GO to better understand the functions and characteristics of identified mRNA signatures of three ABs (*p* < 0.05). GO analysis were classified into biological process (BP), cellular component (CC) and molecular function (MF), also showed remarkable differences among different groups (Figure S3). For ncRNAs, 20,889 lncRNAs, 640 miRNAs and 769 circRNAs were detected and the expression profiles were presented in clustered heatmaps and scatter plots (Figure 2A, B Figure S4A, B, E, F). VENN analysis was performed to screen out ncRNA signatures of three ABs followed by hierarchical clustering (Figure 2C, D; Figure S4C, D, G, H). These ncRNA signatures identified in different ABs may serve as potential biomarkers for identification of ABs categories. We then divided the differentially expressed RNAs into upregulated RNAs and downregulated RNAs, and analyzed the proportion of differentially expressed RNAs in each comparison group (Figure S5). Results showed that mRNA and lncRNA account for more than 95% of the total RNA cargo; however, the mRNA in ABs reflects more about the gene expression pattern of parental cells instead of their own function as mRNA in ABs is rarely translated 36. LncRNA, on the other hand, has been shown to serve profound regulatory roles through vesicle transportation 37. In this regard, we focused on lncRNAs to understand the biological functions of osteoclast derived ABs. Collectively, we mapped the transcriptome and small RNA profiling of BMM-ABs, pOC-ABs and mOC-ABs in detail. Besides, bioinformatic analysis revealed a clearly distinct function of these ABs based on their different genetic contents. We previously reported the genomic and functional difference of osteoclasts at different stages on a cellular level 26. Similar conclusion was obtained from the ABs derived from correspondent parental cell. We then ask if there is a biological connection between ABs and their parental cells.

### Transcriptional similarities of osteoclast derived ABs and corresponding parental cells

**Figure 3:**
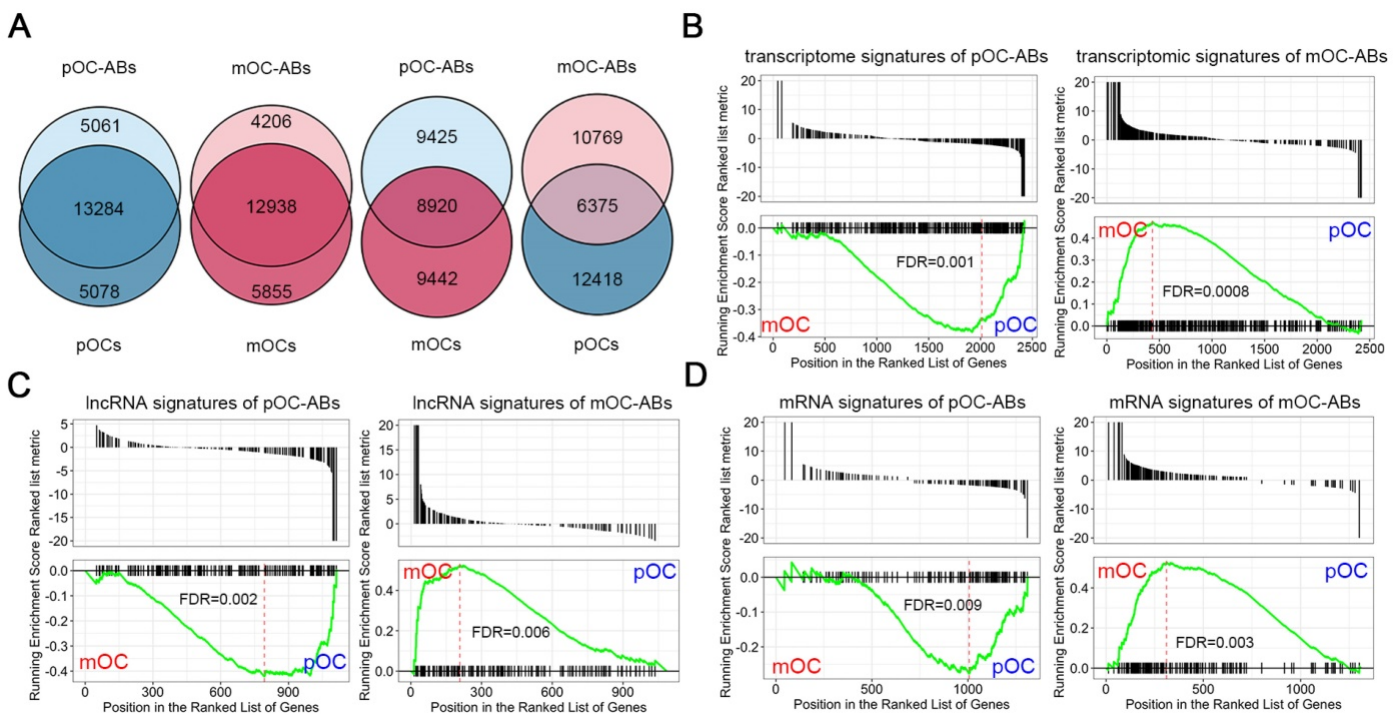
** Transcriptional similarities of osteoclast derived ABs with their parental cells.** (**A**) Venn diagram comparing all identified RNAs of the pOC-ABs and mOC-ABs with corresponding parental cell or non-parental cell. Gene set enrichment analysis (GSEA) plots showing the correlation of pOC-ABs and mOC-ABs (**B**) transcriptome signatures, (**C**) lncRNA signatures and (**D**) mRNA signatures with genes expressed in pOC or mOC. The RNA signatures of pOC-ABs preferred to show a pOC-phenotype, while signatures of mOC-ABs preferred to display a mOC-phenotype.

To compare the transcriptome of osteoclast derived ABs with corresponding parental cells (pOC and mOC), we performed VENN analysis of two data sets for all identified RNAs between ABs and the corresponding parental cells. Results indicated the approximately 75% overlaps between the whole transcriptomes of ABs and their parental cells, whereas the overlaps between ABs and non-parental cells were only less than 40% (Figure 3A). Then, we performed gene set enrichment analysis (GSEA) to search the correlations between RNA signatures of ABs and the parent cells by using our previously described gene expression data of BMMs, pOC and mOC 26. Similarly, pOC-AB transcriptomic signatures bore resemblance to a pOC-like phenotype, while mOC-AB better exhibited a mOC-like phenotype (Figure 3B). It has been demonstrated that the vesicles carrying lncRNAs are well recognized for their regulatory roles 37,38. Using GSEA, we further identified that lncRNA signatures of ABs also shared high similarities with the parental cells (Figure 3C). We also identified the mRNA signature similarity of ABs with the parental cells (Figure 3D). Together, we demonstrated that pOC-ABs and mOC-ABs exhibited a high degree of similarity to the parental cell pOC and mOC on the transcriptomic level.

### Functional similarities of ABs with parental osteoclasts are mediated by key lncRNAs

**Figure 4:**
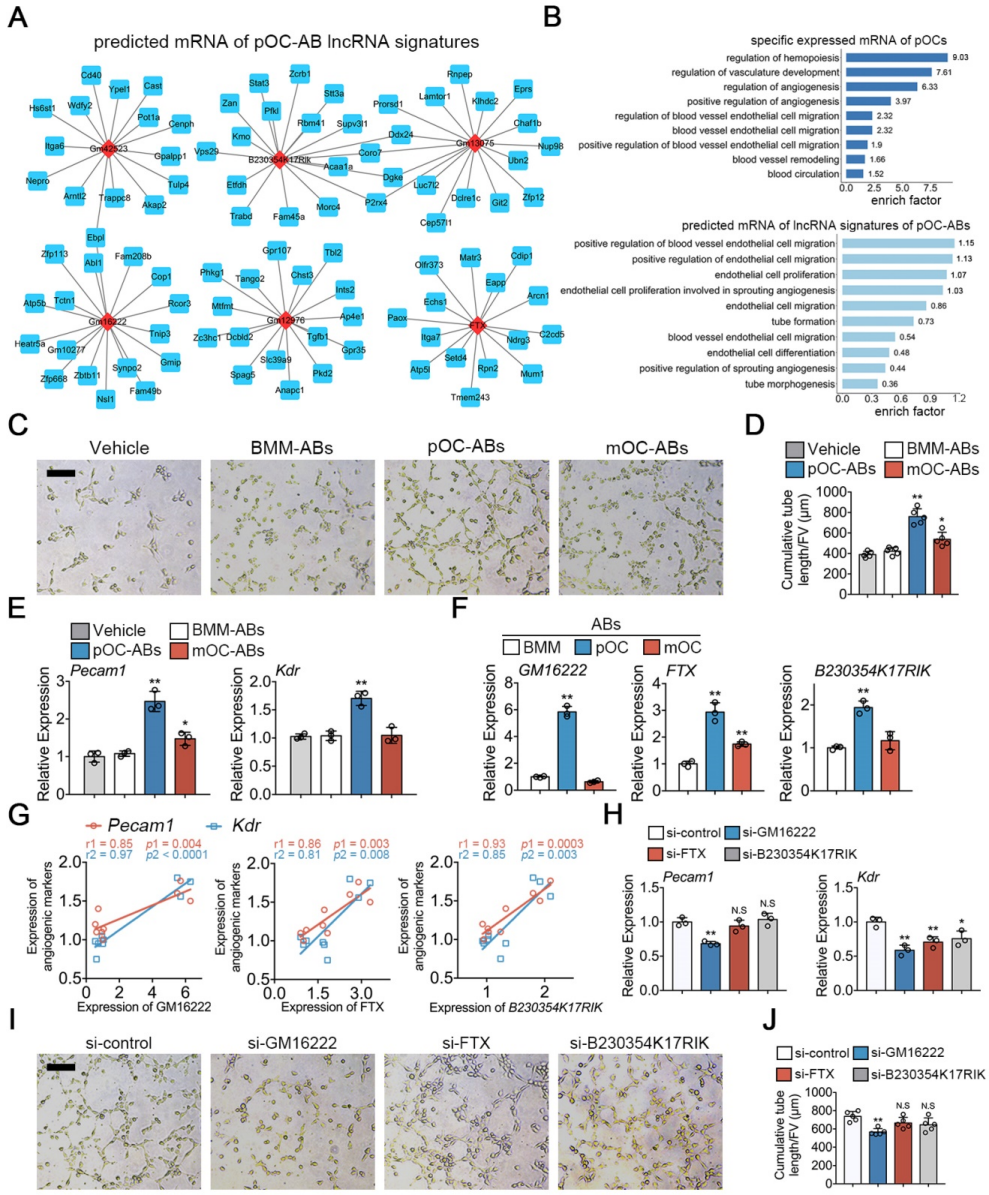
** Functional similarities of pOC-ABs with the parent cell pOCs.** (**A**) Construction of the lncRNA-mRNA interaction network for top 6 most differentially expressed lncRNAs of pOC-ABs. Rhombic red nodes represent lncRNAs and square blue nodes represent mRNAs. (**B**) Angiogenesis related BP terms were shown by using GO enrichment analysis for specific expressed mRNA of pOCs or predicted target mRNAs of lncRNA signatures of pOC-ABs. (**C**) Representative Matrigel tube formation assay images of EPCs cultured with different AB-media. Bar represents 100 µm. (**D**) Quantitative analysis of cumulative tube length in (C), n = 5 per group. (**E**) Relative mRNA expression levels of *Pecam1* and *Kdr* in EPCs cultured with different AB-media. (**F**) Relative expression levels of lncRNA *GM16222*, *FTX* and *B230354K17RIK* in pOC-ABs. (**G**) Correlations between lncRNA *GM16222*, *FTX* and *B230354K17RIK* with* Pecam1* and *Kdr*. (**H**) Relative mRNA expression levels of *Pecam1* and *Kdr* in EPCs cultured with pOC-AB-media with indicated treatments. (**I**) Representative Matrigel tube formation assay images of EPCs cultured with three lncRNA-knockdown pOC-ABs. Bar represents 100 µm. (**J**) Quantitative analysis of cumulative tube length in (I). Images are representative of n = 5 independent experiments. The data in the figures represent the averages ± SD. Significant differences are indicated as * (*p* < 0.05) or ** (*p* < 0.01) paired using Student's t test unless otherwise specified.

To investigate if the transcriptional similarities between ABs with corresponding parental cells may extend to the functional level, we first used cis and trans relationship to predict the target mRNAs of lncRNAs 39 in pOC-ABs. Top 6 most differentially expressed lncRNAs (marked in red) and corresponding predicted interacting mRNAs (marked in blue) were shown in different sub-networks (Figure 4A). We further found that these vesicular lncRNA linked mRNAs were highly enriched in angiogenesis related BP terms, which indicated that pOC-ABs are potentially pro-angiogenic similar with the parental cell (Figure 4B). Among which, targeting genes of both lncRNA *GM16222*, *FTX* and *B230354K17RIK* were found to include *Pecam1* (encoded for platelet/endothelial cell adhesion molecule 1 or CD31), *Kdr* (encoded for vascular endothelial growth factor receptor 2), and *TGFβ1* (Figure S6A). Our previous study has found that ABs can be engulfed by recipient cells and subsequently regulate their cell differentiation through signal transduction 25. Hence, we generated AB-media by adding cell-free ABs derived from BMM, pOC and mOC to the complete medium of endothelial progenitor cells (EPCs) to further examine whether ABs have an impact on angiogenesis (Figure 4C). Consistent with our bioinformatics predictions, the cumulative tube length formed by EPCs was remarkably longer when incubated with pOC-ABs compared with control media without ABs (Figure 4D). Expression of *Pecam1* and *Kdr* showed significant increase in EPCs cultured with pOC-AB-media (Figure 4E).

The expressions of three lncRNAs were validated by qPCR analysis in parental pOCs (Figure S6C) and pOC-ABs (Figure 4F). Linear regression analysis revealed highly positive correlations between lncRNAs and the pro-angiogenic genes (Figure 4G). We then constructed lentivirus to interfere with the expression of *GM16222*, *FTX* and *B230354K17RIK* (Figure S6E, F). Knockdown of *GM16222* in pOCs resulted in the abrogation of pro-angiogenic genes upregulation (Figure 4H). EPCs were then cultured using relative lncRNA-knockdown pOC-AB-media for tube formation assay (Figure 4I). Quantitative analysis showed that *GM16222* knockdown among the three lncRNAs significantly reduced the cumulative tube length, whereas no significant change in cumulative tube length was observed after *FTX* and *B230354K17RIK* knockdown (Figure 4J).

**Figure 5:**
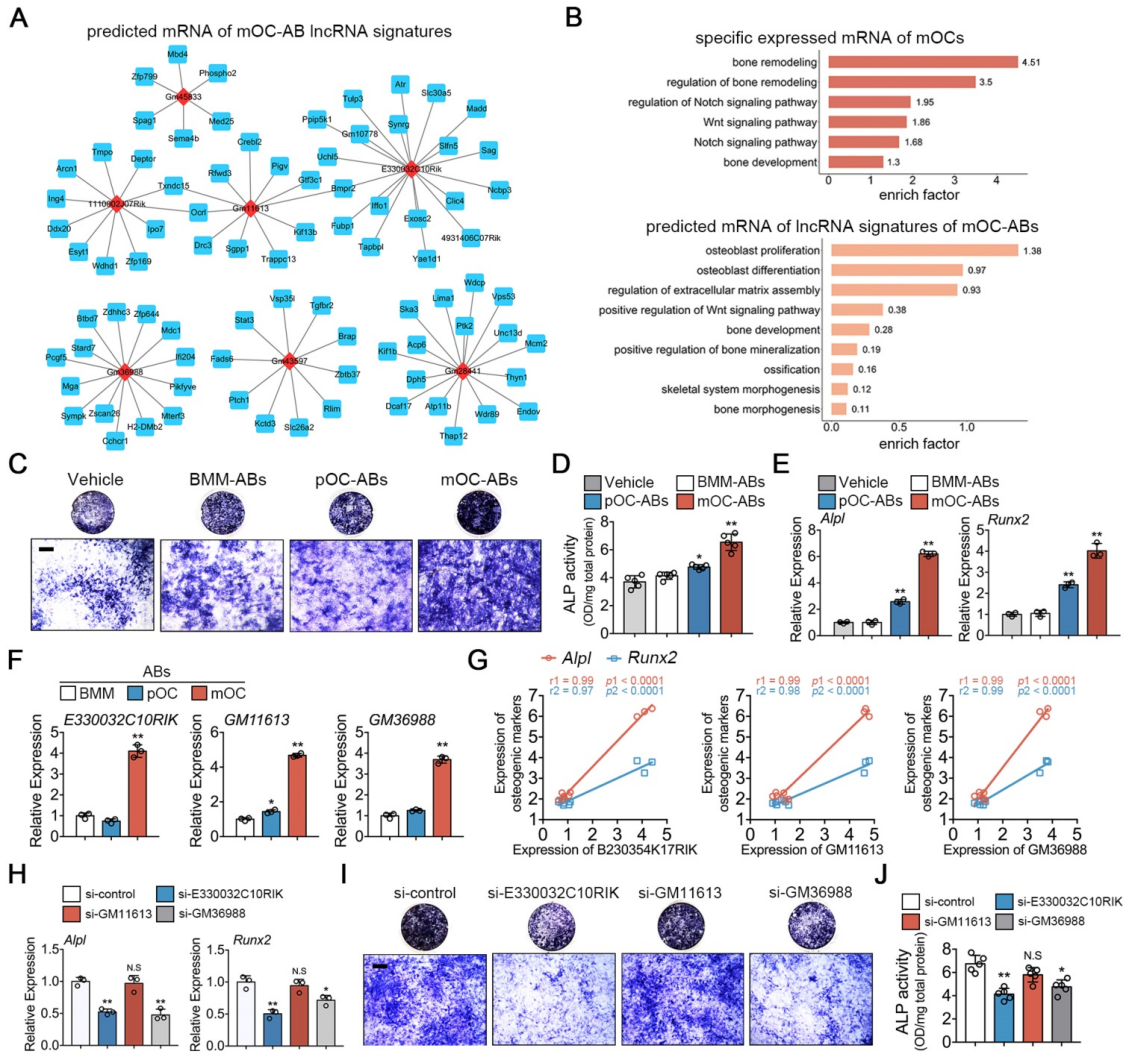
** Functional similarities of mOC-ABs with the parent cell mOCs.** (**A**) Construction of the lncRNA-mRNA interaction network for top 6 most differentially expressed lncRNAs of mOC-ABs. Rhombic red nodes represent lncRNAs and square blue nodes represent mRNAs. (**B**) Osteogenesis related BP terms were shown by using GO enrichment analysis for specific expressed mRNA of mOCs or predicted target mRNAs of lnRNA signatures of mOC-ABs. (**C**) Representative images of ALP staining of MC3T3-E1 cells cultured with different AB-media. Bar represents 200 µm. (**D**) Quantitative analysis of ALP activity of MC3T3-E1 cells cultured with different ABs, n = 5 per group. (**E**) Relative mRNA expression levels of *Alpl* and *Runx2* in MC3T3-E1 cells cultured with different AB-media. (**F**) Relative expression levels of lncRNA *E330032C10RIK*, *GM11613* and *GM36988* in mOC-ABs. (**G**) Correlations between lncRNA *E330032C10RIK*, *GM11613* and *GM36988* with* Alpl* and *Runx2*. (**H**) Relative mRNA expression levels of *Alpl* and *Runx2* in MC3T3-E1 cells cultured with mOC-AB-media with indicated treatments. (**I**) Representative images of ALP staining of MC3T3-E1 cells cultured with three lncRNA-knockdown mOC-ABs. Bar represents 200 µm. (**J**) Quantification of ALP activity of MC3T3-E1 cells in (I). Images are representative of n = 5 independent experiments. The data in the figures represent the averages ± SD. Significant differences are indicated as * (*p* < 0.05) or ** (*p* < 0.01) paired using Student's t test unless otherwise specified.

Likewise, we predicted the target mRNAs of lncRNA signatures in mOC-ABs and constructed the lncRNA-mRNA prediction network of top 7 most differentially expressed lncRNAs in mOC-ABs (Figure 5A). GO enrichment analysis was performed on the specific expressed mRNAs in mOCs and predicted target mRNAs of lncRNA signatures in mOC-ABs revealing high enrichment related with osteogenesis (Figure 5B). We found three lncRNAs *E330032C10RIK*, *GM11613* and *GM36988*, with their potential targeting genes all include *Alpl* (encoded for alkaline phosphatase), *Runx2* (encoded for runt related transcription factor 2), and *Mef2c* (Figure S6B). Preosteoblastic MC3T3-E1 cells cultured with AB-media revealed that mOC-AB specifically increased the ALP activity, and *Alpl*, *Runx2* expression (Figure 5C-E). Quantitative PCR analysis was performed validating the high expression of these three lncRNAs in parental mOC (Figure S6D) and mOC-ABs (Figure 5F). Linear regression analysis revealed highly positive correlations between lncRNAs and the pro-osteogenic genes (Figure 5G). We then constructed lentivirus interfering with the expression of *E330032C10RIK*, *GM11613* and *GM36988* (Figure S6E, G). Knockdown of *E330032C10RIK* and *GM36988* in mOCs resulted in the abrogation of pro-osteogenic genes upregulation (Figure 5H). Corresponding lncRNA-knockdown mOC-ABs were co-cultured with MC3T3-E1 cells for ALP and Alizarin red stain (Figure 5I, Figure S6H). Quantitative analysis showed that knockdown of *E330032C10RIK* and *GM36988* significantly decreased ALP activity and mineralization, whereas no significant difference was observed after *GM11613* knockdown (Figure 5J). Collectively, we demonstrated the important role of key lncRNAs in AB-involved angiogenic and osteogenic activities. These key lncRNAs determined the biological roles of ABs in angiogenesis and osteogenesis, deletion of these key lncRNAs resulted in diminished or loss of corresponding functions.

We also explored deeper trying to understand the underlying molecular mechanism of the AB derived lncRNA effects. *In vitro* results suggested that both lncRNA *GM16222* and *E330032C10RIK* may serve as competing endogenous RNA (ceRNA) that bind with relative miRNAs regulating cell differentiation (Figure S7, S8). Detailed description is included in the supplementary text.

### pOC-ABs induce angiogenesis and mOC-ABs promote osteogenesis *in vivo*

**Figure 6:**
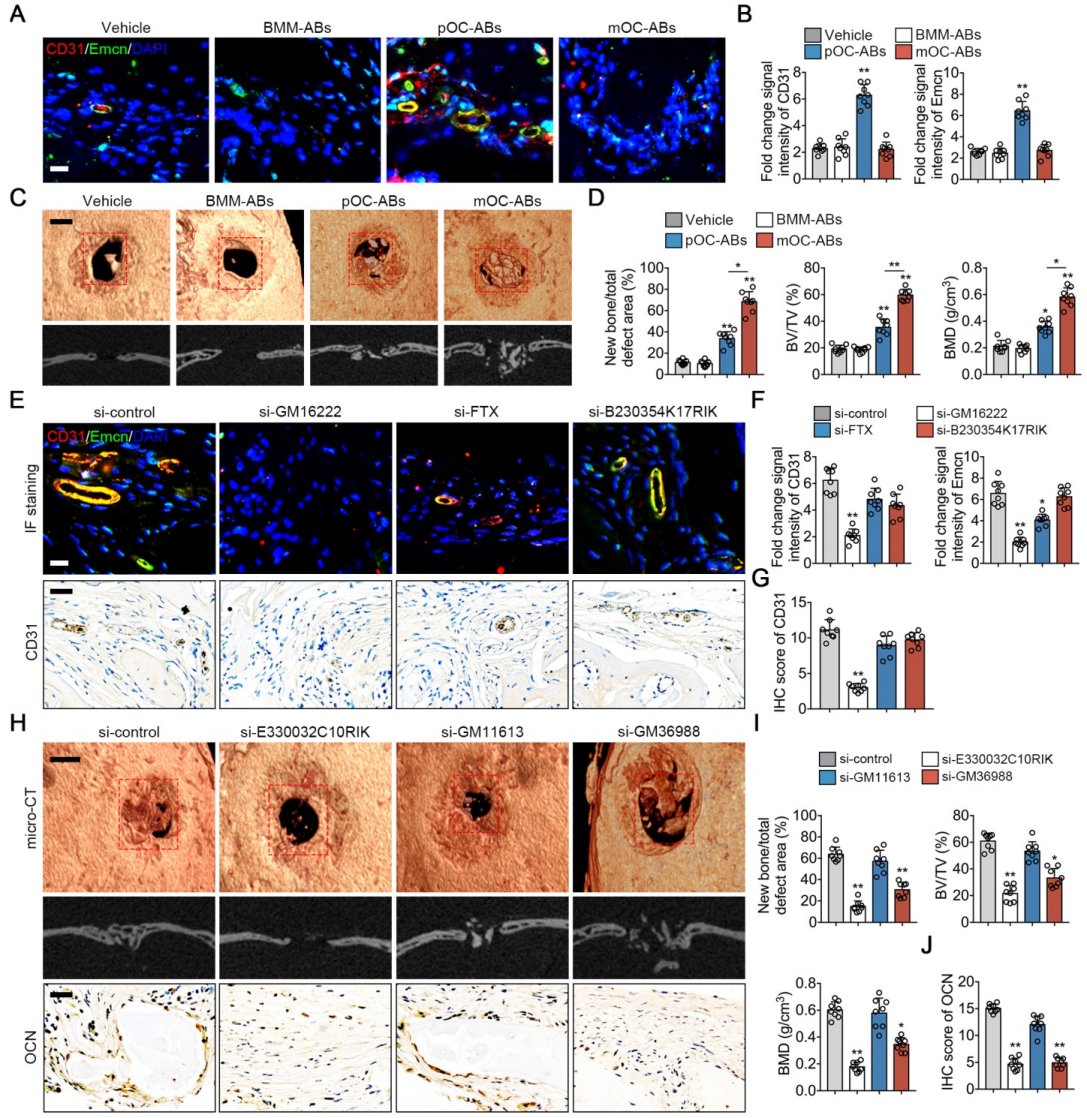
***In vivo* bone regeneration effects of osteoclast derived ABs.** (**A**) Representative images of immunostaining of CD31 (red) and Emcn (green) of mice grafted with AB-DBM for 2 weeks. Bar represent 10 µm. (**B**) Quantification of relative CD31 and Emcn immunostaining intensities in indicated groups, n = 8 per group. (**C**) Representative general micro-CT images (upper) and coronal micro-CT images (lower) of mice grafted with AB-DBM for 4 weeks. Bar represent 2 mm. (**D**) Quantitative micro-CT analysis showed the amount of bone formation, bone volume density (BV/TV), and bone mineral density (BMD) of total defect repair area in indicated groups. mOC-AB-DBM displayed the best osteogenic mineralization at 4 weeks, n = 8 per group. (**E**) Representative images of immunostaining of CD31 (red) and Emcn (green), micro-CT images and IHC staining of CD31 of mice grafted with relative lncRNA knockdown pOC-AB-DBM for 2 weeks. Bar represent 10 µm in immunostaining and 50 µm in IHC staining. (**F**) Quantification of relative CD31 and Emcn immunostaining intensities in indicated groups, n = 8 per group. (**G**) Semi-quantitative analysis of CD31 in indicated groups, n = 8 per group. (**H**) Representative images of micro-CT and IHC staining of OCN of mice grafted with relative lncRNA knockdown mOC-AB-DBM for 4 weeks. Bar represent 2 mm in micro-CT images and 50 µm in IHC staining. (**I**) Quantitative micro-CT analysis showed the amount of bone formation, BV/TV, and BMD of total defect repair area in indicated groups, n = 8 per group. (**J**) Semi-quantitative analysis of OCN in indicated groups, n = 8 per group. The data in the figures represent the averages ± SD. Significant differences are indicated as * (*p* < 0.05) or ** (*p* < 0.01) paired using Student's t test unless otherwise specified.

To confirm the functional similarities of ABs with parental cells *in vivo*, we generated calvarial defect mice using cranial drilling. This mouse model is useful to evaluate the effects of cells- or EVs-modified scaffolds on angiogenesis and osteogenesis 40,41. DBM pre-incubated with different ABs was grafted in the defect area followed by normal breeding for two weeks (for angiogenesis evaluation) or four weeks (for osteogenesis evaluation) before euthanasia. Previous studies identified a distinctive subtype of vessel known as type H vessel that strongly positive for CD31 and endomucin (CD31^hi^Emcn^hi^), was demonstrated to couple angiogenesis and osteogenesis during bone remodeling 23,42. Here we performed immunostaining of CD31 and Emcn to explore the impact of ABs on type H vessel formation (Figure 6A). Quantitative analysis showed that fold change signal intensities of CD31 and Emcn of mice grafted with pOC-AB-DBMs was the highest suggesting the promoting effect of pOC-ABs on angiogenesis (Figure 6B). For more specific osteogenic potency evaluation, bone regeneration was evaluated by micro-CT four weeks after implantation of DBM incubated with different ABs (Figure 6C). Quantitative analysis confirmed that both pOC-ABs and mOC-ABs enriched DBM grafting significantly increased bone regeneration, bone volume density (BV/TV) and bone mineral density (BMD), whereas mice treated with mOC-ABs showed the highest osteogenic potency (Figure 6D). We then performed H&E and TRAP stain of the calvarial section, results showed that both osteoclast and osteoblast number were increased by AB-DBM grafting (Figure S9). The above data demonstrated the pro-angiogenic ability of pOC-ABs and the osteogenic potential of mOC-ABs, which is consistent with *in vitro* experiment. We next evaluated the impact of key lncRNAs knockdown of ABs on angiogenesis and bone regeneration in the defect repair area. Intriguingly, knockdown of *GM16222* significantly abolished the enhancement of pOC-ABs in angiogenesis revealing the essential role of *GM16222* in pOC-ABs induced angiogenesis marked by decreased CD31^hi^Emcn^hi^ cell formation and overall CD31 (Figure 6E-G). For osteogenic evaluation, corresponding lncRNA-knockdown mOC-ABs were grafted in defect area and after four weeks, bone regeneration was detected using micro-CT and IHC of OCN (Figure 6H). Quantitative analysis showed that knockdown of both *E330032C10RIK* and *GM36988* in mOC-ABs resulted in the abrogation of bone regeneration marked by decreased new bone formation rate and OCN score (Figure 6I, J). So far, we demonstrated the function similarities of ABs with parental cells in a murine model of calvarial bone defect. The inherited biological functions of ABs were shown determined by several enriched lncRNAs of which the knockdown abolished AB functions.

## Discussion

In this study, we investigated the whole transcriptome paralleled with small RNA profile in AB contents using osteoclast as a cell model. Stimulated by M-CSF and RANKL, pOC and mOC seems only different regards to the induction time. However, both cellular morphology function and regulatory roles changed dramatically 43, making this cell type a good model to study the content changes of ABs derived from a same cell type at different stages. We already have the knowledge that AB contents are specific among different cell types 44, our results further showed that AB transcriptome signature remains specific even in the same cell type at distinct differentiation stages. However, the transcriptome profiles of ABs are highly similar with their respective parental cells suggesting a similar biological function. For verification, *in vitro* studies were performed detecting whether ABs share the most specific regulatory function of their parental cells. Interestingly, through several key lncRNAs, pOC-ABs inherited the pro-angiogenesis activity of pOCs while mOC-ABs inherited the pro-osteogenesis activity of mOCs. We concluded that the inheritance of ABs from the parental cells can be detected both on the genetic and functional levels. In our study, we focused on angiogenesis and osteogenesis, which are highly intertwined during bone formation. It has been characterized that a bone specific CD31^high^Emcn^high^ endothelial cell type mediates growth of the bone vasculature, generate distinct metabolic and molecular microenvironments 42,45. Non resorbing osteoclast as well as pOC, are both reported facilitating CD31^high^Emcn^high^ endothelial cell formation via secretion of pro-angiogenic factors 23,46.

A very recent study reported that apoptotic endothelial cells release small EVs loaded with immunostimulatory viral-like RNAs 47. It is interesting to notice that upon apoptotic stimulation, endothelial cells generate both ABs and exosome-like nanovesicles (ApoExos); only ApoExos are immunogenic and cause inflammation in mice. The authors also claimed that the RNA contents in ABs are more similar with parental endothelial cells which supported our data. Apart from the AB RNA content similarity with the parental cells demonstrated by our results, the RNA cargo in the exosomes and microvesicles also showed cell type-specific expression patterns. In another whole-transcriptome RNA-seq study, the authors showed that human mast and erythroleukemic cell lines release two distinct exosomes populations 48. Their results also suggested that cells can release multiple types of extracellular RNA with substantial differences in RNA species content. Although both EVs showed cell type-specific expression patterns in RNA cargos, a study showed that exosomes contained predominantly small RNA and much less rRNA as compared to both ABs and microvesicles 49. Our study of osteoclast derived ABs showed that instead of small RNAs, lncRNAs and mRNAs are the dominant RNA cargo (more than 95%) in these vesicles. This specific RNA loading from live cells to extracellular vesicles reveals the possibility that exosome secretion has more specific biological aims, while ABs and microvesicles RNA signature reflected a more comprehensive picture of the total cell content. Both AB mediated cell coupling and other EV mediated cell coupling may exist at the same time in bone homeostasis. However, we assume AB mediated osteoclast-osteoblast coupling involved more in bridging bone resorption and formation, as osteoclast lifespan is accordant with the cycle of bone remodeling. The periodical cell apoptosis and generation of ABs provided the basis for signal releasing in transiting phases during bone turnover.

Previous findings regarding the biological functions of ABs have been focused on their immunomodulatory roles. It has been recognized that impaired clearance of dying cells and the relevant ABs contribute to the development of autoantibodies in autoimmune conditions 50-52. In HIV infection, it has been shown that ABs generation by host cells were able to modulate the dendritic cells response via binding to the CD44 receptor, resulting in decrease cytokine production from dendritic cells and inhibition of their ability to prime T cells or natural killer cells 53. It is worth to notice that tumor antigen PMEL was found in apoptotic vesicles 54 and mice immunized with B16-F1 melanoma cell derived apoptotic vesicles showed increased antitumor immunity against subsequent tumor challenges 55. In addition to the presentation of self-antigens, it is important to note that under conditions wherein infected cells undergo apoptosis, the resultant apoptotic vesicles may also harbor antigens from the infectious agent 56. The surface marker and protein cargos of ABs have been widely studied, whereas the AB RNA contents and their potential regulatory roles are rarely reported. Tumor cell derived exosomes and MVs are considered as critical mediators of intracellular communication between tumor cells and neighboring non-tumor cells 57. It has been well recognized that tumor cells can secrete high levels of potent angiogenic factors which contribute to the tumor invasion and are associated with poor prognosis 58,59. Studies also reported that tumor-derived exosomes containing TGF-β convert fibroblasts into myofibroblasts, which contribute to tumor angiogenesis, growth and local invasion 60,61. Besides, exosomes and MVs generated by breast cancer cells can promote vascular leakiness through S100 proteins and miR-105 thereby facilitating circulating tumor cells arrival to distant sites 62,63. Cianciaruso et al. reported that tumor associated macrophages derived EVs displayed proteomic characteristics of M1 macrophages and induced T cell proliferation and activation 64. In our study, we demonstrated that AB from osteoclasts also have similar transcriptional profiles and functions with their parental cells, revealing that the functional similarity between EVs and parental cells is not accidental. In addition, it is worthy to further explore the involvement of osteoclast derived ABs in primary and metastatic bone tumors. Osteoclasts as myeloid-derived suppressor cells (MDSCs) participated in various bone tumor metastasis 65. Considering the over activation of osteoclast in several particular tumor bone metastasis, it is also interesting to test if the ABs derived from those osteoclasts might function as suppressor factors for other immune cells.

In conclusion, our study mapped the whole transcriptome and small RNA profiles of osteoclast derived ABs. We demonstrated that ABs derived from osteoclasts have similar genetic phenotype with the corresponding parental cells, of which the similarities extend to functional level through several key lncRNAs. The data indicated a functional inheritance of osteoclast derived ABs from the parental cells suggesting their bridging role in osteoclast-osteoblast coupling in bone remodeling.

## Supplementary Material

Supplementary figures and tables.
