# Supplementary material for: Osteoclast-derived apoptotic bodies show extended biological effects of parental cell in promoting bone defect healing
Source: Theranostics. 2020 May 22;10(15):6825–38. doi: 10.7150/thno.45170 (PMC7295057; doi:10.7150/thno.45170)
Supplement: Supplementary file 1 — Supplementary figures and tables. [file thnov10p6825s1.pdf]

Osteoclast-derived apoptotic bodies show extended biological effects of parental cell in promoting bone defect healing

Qinyu Ma [1], Mengmeng Liang [2], Nathachit Limjunyawong [3], Yang Dan [4], Junchao Xing [1], Jianmei Li [2], Jianzhong Xu [1]*, and Ce Dou [1,5]*

[1] Department of Orthopedics, Southwest Hospital, Third Military Medical University, Chongqing 400038, China.

[2] Department of Biomedical Materials Science, Third Military Medical University, Chongqing 400038, China.

[3] The Solomon H. Snyder Department of Neuroscience, Johns Hopkins University School of Medicine, Baltimore, Maryland 21205, USA.

[4] Department of Orthopedics, Union Hospital,Tongji Medical College, Huazhong University of Science and Technology, Wuhan 430022, China.

[5] Department of Orthopedic Surgery, Johns Hopkins University School of Medicine, Baltimore, Maryland 21205, USA.

Corresponding author:

Jianzhong Xu, Department of Orthopedics, Southwest Hospital, Third Military Medical University, Gaotanyan Street No.30, Chongqing 400038, China. Email: xujianzhong1962@163.com

Ce Dou, Department of Orthopedics, Southwest Hospital, Third Military Medical University, Gaotanyan Street No.30, Chongqing 400038, China. Email: lance.douce@gmail.com

Keywords: Apoptotic body, Osteoclast, Vesicle bioinformatics, Functional inheritance.

Supplementary Methods and Materials

Cell reagents

Mouse bone marrow cells were isolated and incubated with M-CSF (50 ng/ml) for 24 h. Adherent cells were collected and cultured with M-CSF (50 ng/mL) for another 48-72 h to obtain BMM cells. The endothelial progenitor cells (EPCs) were separated from the bone marrow mononuclear cells (BMPCs) of C57BL/6 mice. Alpha minimal essential Medium (α-MEM) and fetal bovine serum (FBS) were purchased from Gibco (life technologies, USA). Penicillin-streptomycin solution was obtained from Hyclone (Thermo Scientific, USA). Recombinant Mouse RANKL and Recombinant Mouse M-CSF were obtained from R&D Systems (Minneapolis, MN). Antibodies against H3 (bs-17422R), H2B (bs-15400R), C1QC (bs-11337R), C3B (bs-4873R) and GAPDH (bs-0755R) were all purchased from Bioss Antibodies (Beijing, China). TRAP stain kit was obtained from Sigma-Aldrich (NY, USA). ALP stain kit was purchased from Beyotime Biotechnology (Shanghai, China). Matrigel was obtained from BD Biosciences (San Jose, CA, USA). Alizarin stain kit was obtained from Solarbio Life Sciences (Beijing, China).

Flow cytometry

Here we conducted flow cytometric analysis using a BD Accuri C6 flow cytometer, counting 20000 events. After marking with AnnexinV-FITC and PI, the remaining binding buffer was removed and AB-sized extracellular vesicles were pelleted by centrifugation at 3000×g for 30 min.

Lentivirus construction and transfection

The interference (si) lentiviral vector and blank control lentivirus were designed and synthesized by Genomeditech (Genomeditech Co. Ltd., Shanghai) to inhibit corresponding lncRNA expression. BLAST searches were performed using the National Center for Biotechnology Information expressed sequence tag database (https://blast.ncbi.nlm.nih.gov/Blast.cgi) to ensure that the siRNA construct only targeted the specific lncRNA. The lentivirus was mixed with equal volumes of MEM containing 10% FBS to infect BMMs, and the value of MOI (Multiplicity of Infection) was 30. After viral incubation for 48 hours, the transduced cells were transfected in a medium containing 5 mg/ml puromycin. The transfected cells were used for culture and experiments.

Alkaline Phosphatase (ALP) and Alizarin red staining

Pre-osteoblasts MC3T3-E1s cells were cultured in α-MEM containing 10% FBS and 1% penicillin/streptomycin. For osteogenesis assays, 5×10^4 cells were seeded in 24-well plates and incubated at 37°C for 24h. Then cultured media was removed and cells were incubated in osteoblast differentiation medium (α-MEM supplemented with 10% FBS, 1% penicillin/streptomycin, 10mM β-Glycerol phosphate disodium salt, 50 μg/mL VC and 100 μM dexamethasone) for 7 days. 7 days later, cells were washed with PBS twice and fixed with 4% paraformaldehyde for 30min at room temperature and then treated according to the protocol by alkaline phosphatase assay kit (Beyotime Biotechnology). ALP activity was measured by colorimetric analysis using the multi-detection microplate reader (BioTec Instruments, Winooski, VT, USA). To assess the deposition of calciumion, cells were fixed in

4% paraformaldehyde and then stained with 1% Alizarin red-S (Solarbio Life Sciences) after 14 days of osteogenic induction.

Immunofluorescent Staining for CD31hiEmcnhi Vessels

To investigate the expression of CD31 and endomucin of newly formed tissue, we performed immunofluorescent staining on the paraffin sections. In short, sections were incubated with individual primary antibodies against CD31 (1:200, Bioss) and endomucin (1:100, Bioss) overnight at 4°C, followed by incubation with secondary antibodies conjugated with fluorescence at room temperature in dark for 1h. A Leica TCS SP8 confocal microscope was used for imaging samples.

Western blot analysis

ABs were lysed in a lysis buffer including 10 mM Tris, pH 7.2, 150 mM NaCl, 5 mM EDTA, 0.1% SDS, 1% Triton X-100, and 1% deoxycholic acid. 20 μg of protein samples were subjected to SDS-PAGE followed by transfer onto PVDF membranes. After blocking in 5% skim milk, membranes were incubated with rabbit antibodies against H3, H2B, C1QC, C3B and GAPDH (1:1000, Bioss, Beijing, China) for 12h at 4 °C followed by an incubation with secondary antibody (1:2000) for an hour. Blots against GAPDH and β-actin served as loading control.

RT-qPCR analysis

Total RNA was isolated using Trizol reagent (Life Technologies). Single-stranded cDNA was prepared from 1 μg of total RNA using reverse transcriptase with oligo-dT primer according the manufacturer's instructions (Promega, USA). Two microlitres of each cDNA was subjected to PCR amplification using specific primers shown in Supplementary Table 1.

Supplementary Text

The ceRNA mechanism of lncRNA *GM16222* and *E330032C10RIK*

To understand how lncRNA *GM16222* is involved in the process of endothelial cell migration and differentiation, we first blasted the transcripts of *GM16222* for potential miRNA interactions and found multiple binding sites of miR-205-3p (Fig. S7A). We further found that endogenous miR-205-3p expression in endothelial cells is decreasing during differentiation in the process of tube formation (Fig. S7B). This negative correlation suggests a potential involvement of miR-205-3p in the process of endothelial cell migration and differentiation. We then constructed GM16222 wild type (WT) and mutated (mut) expression plasmid and cotransfected the plasmids with miR-205-3p mimics into the endothelial cell followed by dual luciferase gene reporter assay (Fig. S7C). The results showed that WT plasmid but not mutated plasmid significantly decreased the luciferase activity suggesting the direct binding of GM16222 and miR-205-3p. The regulation of miR-205-3p by GM16222 was further confirmed by qPCR using siRNA of GM16222 (Fig. S7D). To understand the functioning mechanism of miR-205-3p, we first found that *VEGFA* 3'UTR contains the miRNA reaction element (MRE) of miR-205 (Fig. S7E). Dual luciferase gene reporter assay

was used again to validate the binding relationship between miR-205 and *VEGFA* 3'UTR (Fig. S7F). We found that miR-205 directly bind with the 3'UTR MRE of *VEGFA*. qPCR results showed that VEGFA expression was continuously upregulated during endothelial cell differentiation (Fig. S7G). However, the expression of VEGFA can be hindered by either si-GM16222 or additional miR-205-3p mimics (Fig. S7H, S7I). These results suggested that GM16222 and miR-205-3p work as a ceRNA pair regulating the expression of *VEGFA* during endothelial cell differentiation. Although the mechanism we found might not be the whole picture, the ceRNA mechanism of GM16222/miR-205-3p, at least in part, regulating endothelial cell function.

To understand how lncRNA *E330032C10RIK* is involved in the process of osteogenic differentiation, we first blasted the transcripts of E330032C10RIK for potential miRNA interactions and found multiple binding sites of miR-153-3p (Fig. S8A). We further found that endogenous miR-153-3p expression in MC3T3-E1 cells is decreasing during osteogenic differentiation (Fig. S8B). This negative correlation suggests a potential involvement of miR-153-3p in osteogenic differentiation of MC3T3-E1 cells. We then constructed E330032C10RIK wild type (WT) and mutated (mut) expression plasmid and cotransfected the plasmids with miR-153-3p mimics into the MC3T3-E1 cells followed by dual luciferase gene reporter assay (Fig. S8C). The results showed that WT plasmid but not mutated plasmid significantly decreased the luciferase activity suggesting the direct binding of E330032C10RIK and miR-153-3p. The regulation of miR-153-3p by E330032C10RIK was further confirmed by qPCR using siRNA of E330032C10RIK (Fig. S8D). To understand the functioning mechanism of miR-153-3p, we first found that *RUNX2* 3'UTR contains the miRNA reaction element (MRE) of miR-153 (Fig. S8E). Dual luciferase gene reporter assay was used again to validate the binding relationship between miR-153 and *RUNX2* 3'UTR (Fig. S8F). We found that miR-153 directly bind with the 3'UTR MRE of *RUNX2*. qPCR results showed that *RUNX2* expression was continuously upregulated during osteogenic differentiation of MC3T3-E1 cells (Fig. S8G). However, the expression of *RUNX2* can be hindered by either si-E330032C10RIK or additional miR-153-3p mimics (Fig. S8H, S8I). These results suggested that E330032C10RIK and miR-153-3p work as a ceRNA pair regulating the expression of *RUNX2* during osteogenic differentiation of MC3T3-E1 cells. Although the mechanism we found might not be the whole picture, the ceRNA mechanism of E330032C10RIK/miR-153-3p, at least in part, regulating osteogenic differentiation.

Supplementary Figures

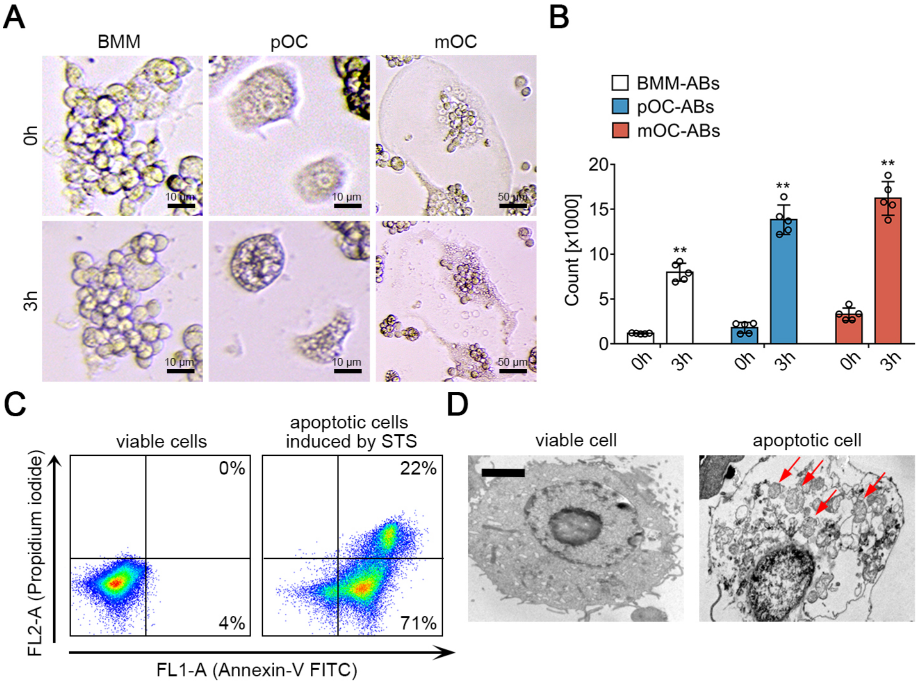

Supplementary Figure S1. Generation and characterization of BMM-ABs, pOC-ABs and mOC-ABs

(A) Apoptosis of BMMs, pOCs and mOCs were induced with STS (0.5 μM) and observed using light microscopy after 3 hour induction. Bar represents 10 or 50 μm as indicated.

(B) Quantification of subcellular fragment counts.

(C) Annexin-V/PI analysis of viable cells and STS induced apoptotic cells. STS-treated cells are mostly positive for Annexin-V (93% of the total), a fraction of which also showed positive for PI (22% of the total).

(D) Representative transmission electron microscopy (TEM) images showing viable and apoptotic osteoclast. Red arrows pointing newly formed apoptotic bodies. Bar represents 2 μm.

The data in the figures represent the averages ± SD. Significant differences are indicated as * ($p < 0.05$) or ** ($p < 0.01$) paired using Student's t test unless otherwise specified.

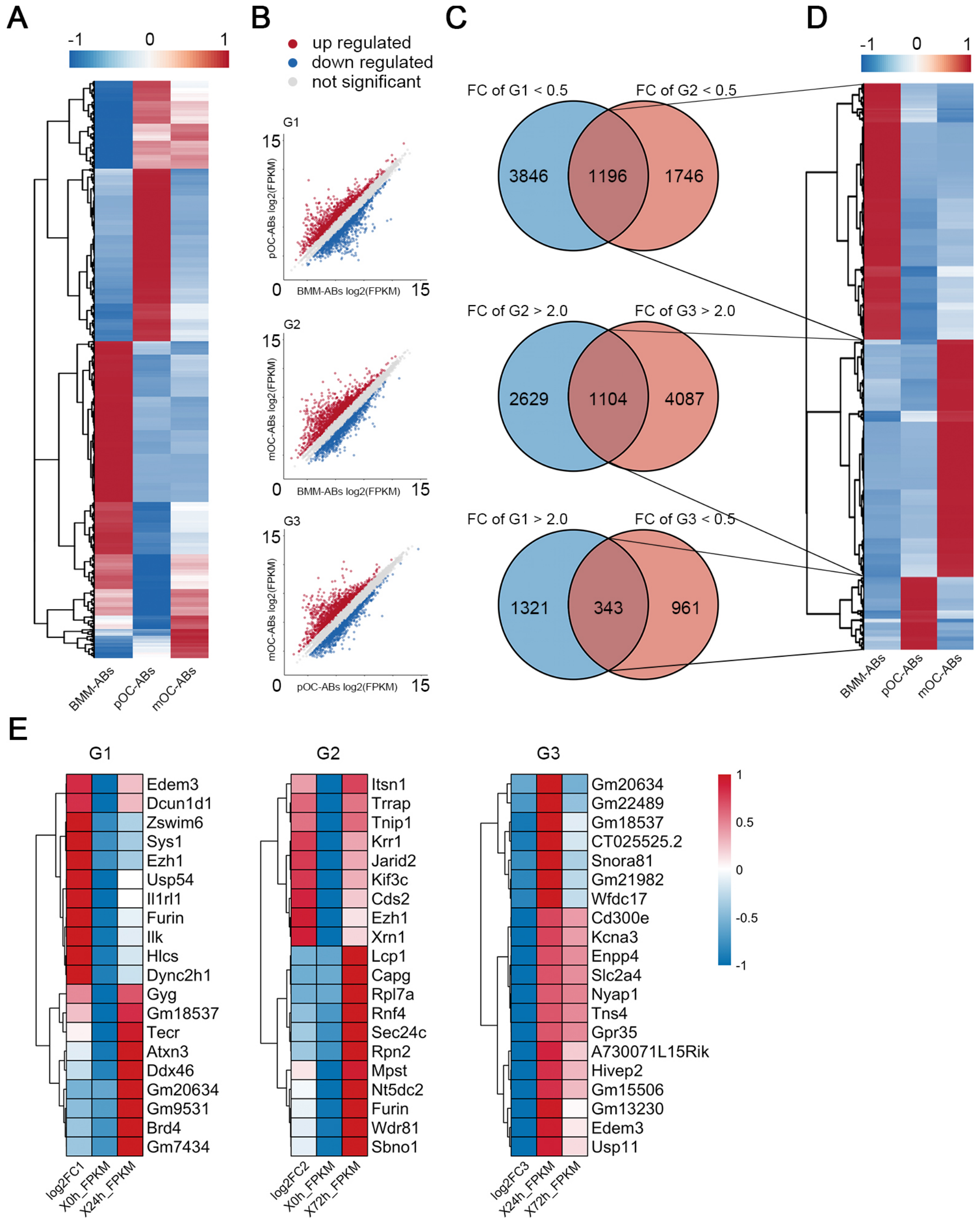

Supplementary Figure S2. mRNA profiling of BMM-ABs, pOC-ABs and mOC-ABs.

(A) Hierarchical clustering of all 14,924 mRNAs expressed in BMM-ABs, pOC-ABs and mOC-ABs.

(B) Scatter plots of all mRNAs expressed in three comparison groups were shown.

(C) VENN analysis screening out mRNA signatures contained in different ABs. Corresponding mRNA signatures were screened out using intersection of three comparison groups G1 (pOC-ABs vs BMM-ABs), G2 (mOC-ABs vs BMM-ABs) and G3 (mOC-ABs vs pOC-ABs). FC, fold change.

(D) Hierarchical clustering of mRNA signatures in three ABs.

(E) Heatmaps showing top 20 mRNAs expressed in three comparison groups.

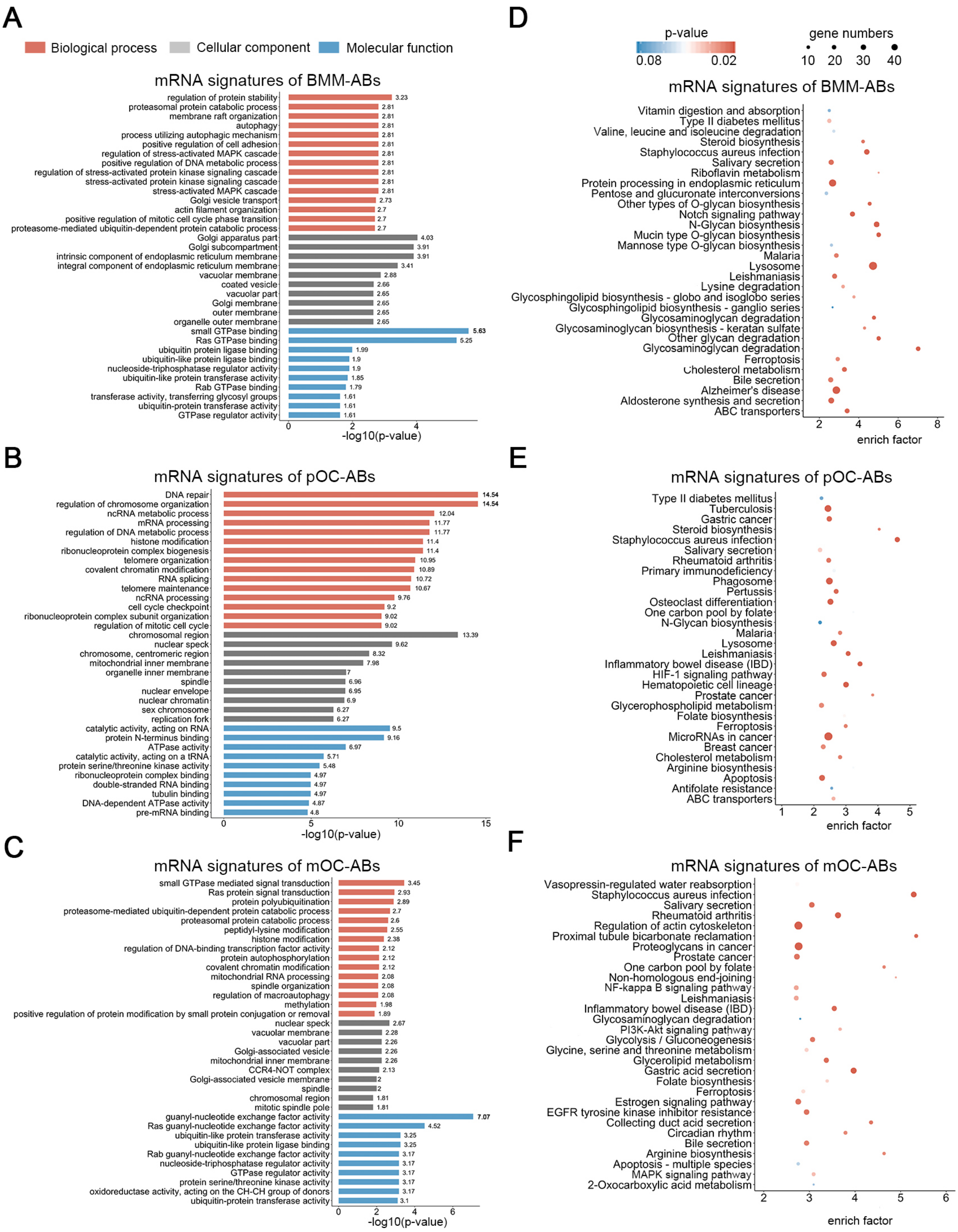

Supplementary Figure S3. Gene ontology (GO) and KEGG pathway enrichment analysis of mRNA signatures.

Top 15 BP, top 10 CC and MF terms for the difference mRNA signatures of (A) BMM-ABs (B) pOC-ABs and (C) mOC-ABs. KEGG pathway enrichment analysis of mRNA signatures of (D) BMM-ABs (E) pOC-ABs and (F) mOC-ABs.

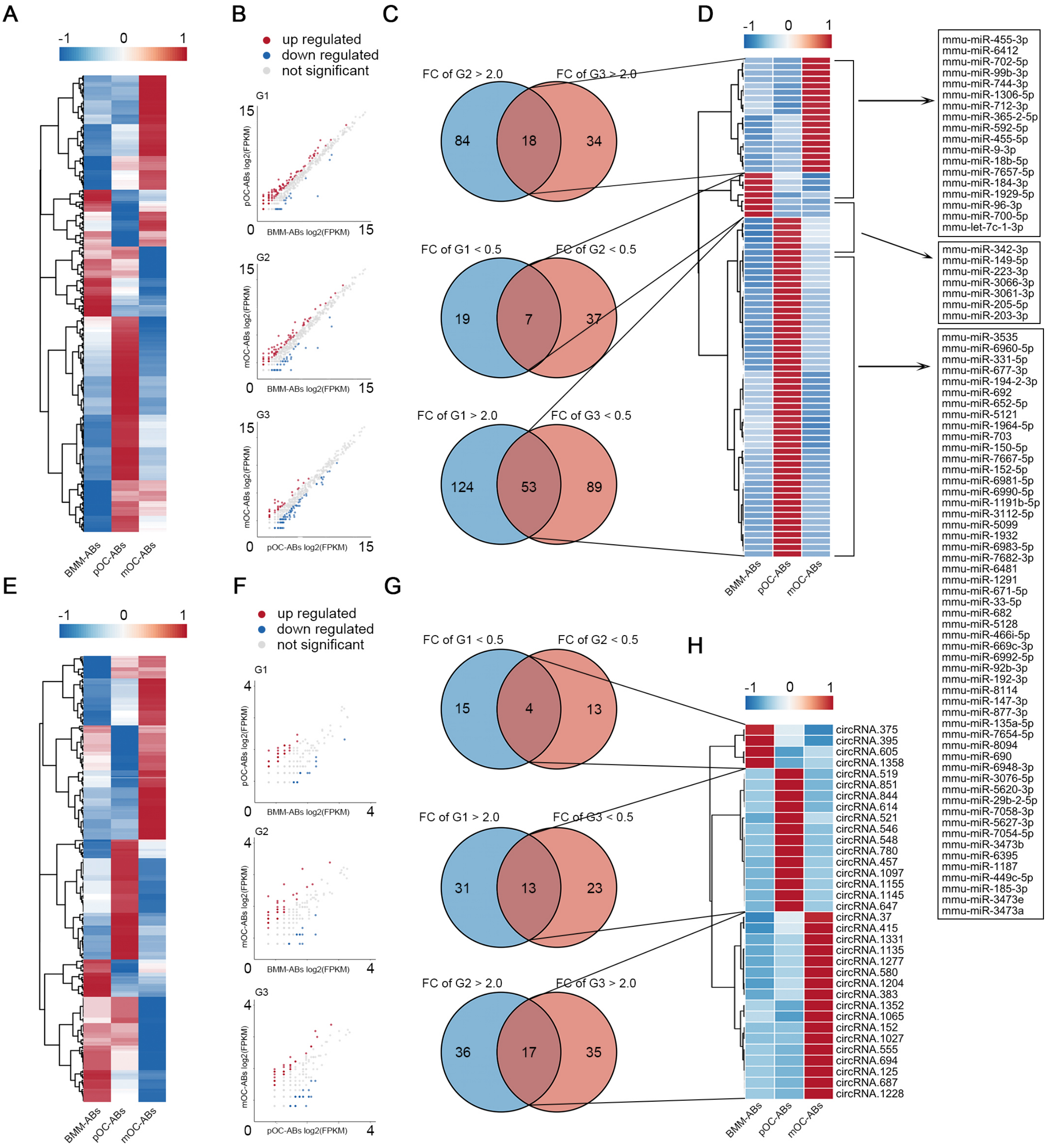

Supplementary Figure S4. miRNA and circRNA profiling of BMM-ABs, pOC-ABs and mOC-ABs.

(A) Hierarchical clustering of all differentially expressed miRNAs in BMM-ABs, pOC-ABs and mOC-ABs.

(B) Scatter plots of differentially expressed miRNAs between three comparisons were shown.

(C) VENN analysis screening out miRNA signatures contained in three ABs.

(D) Hierarchical clustering of miRNA signatures in three ABs.

(E) Hierarchical clustering of all differentially expressed circRNAs in BMM-ABs, pOC-ABs and mOC-ABs.

(F) Scatter plots of differentially expressed circRNAs between three comparisons were shown.

(G) VENN analysis screening out circRNA signatures contained in three ABs.

(H) Hierarchical clustering of circRNA signatures in three ABs.

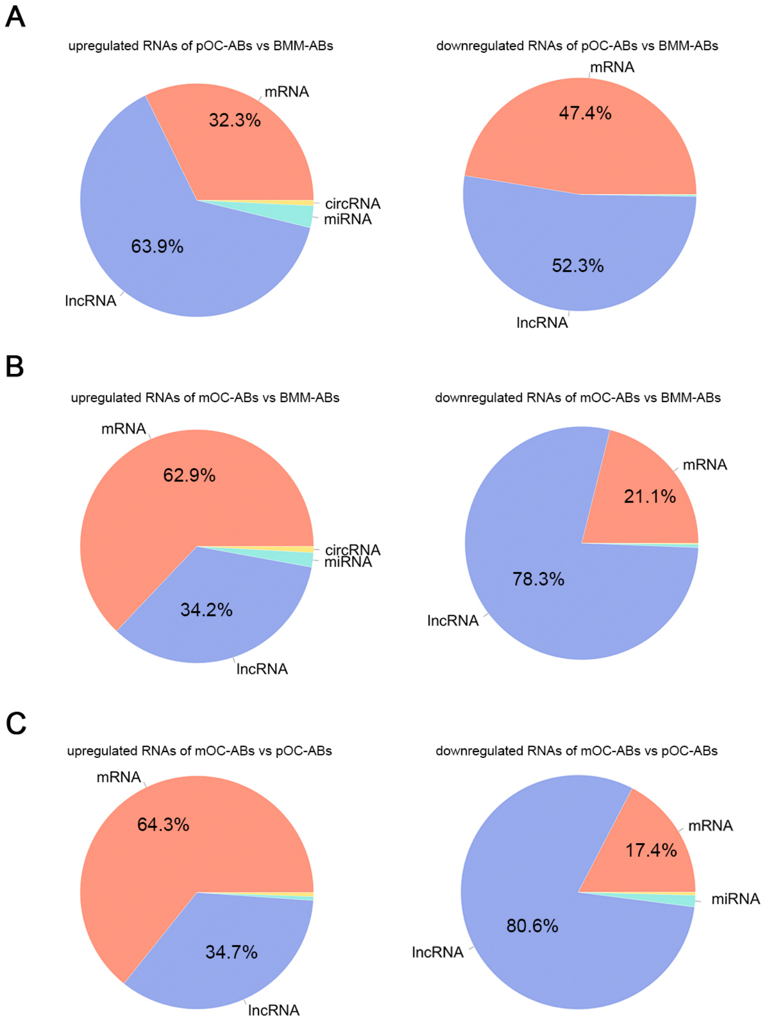

Supplementary Figure S5. The proportion of differentially expressed RNAs in each comparison group

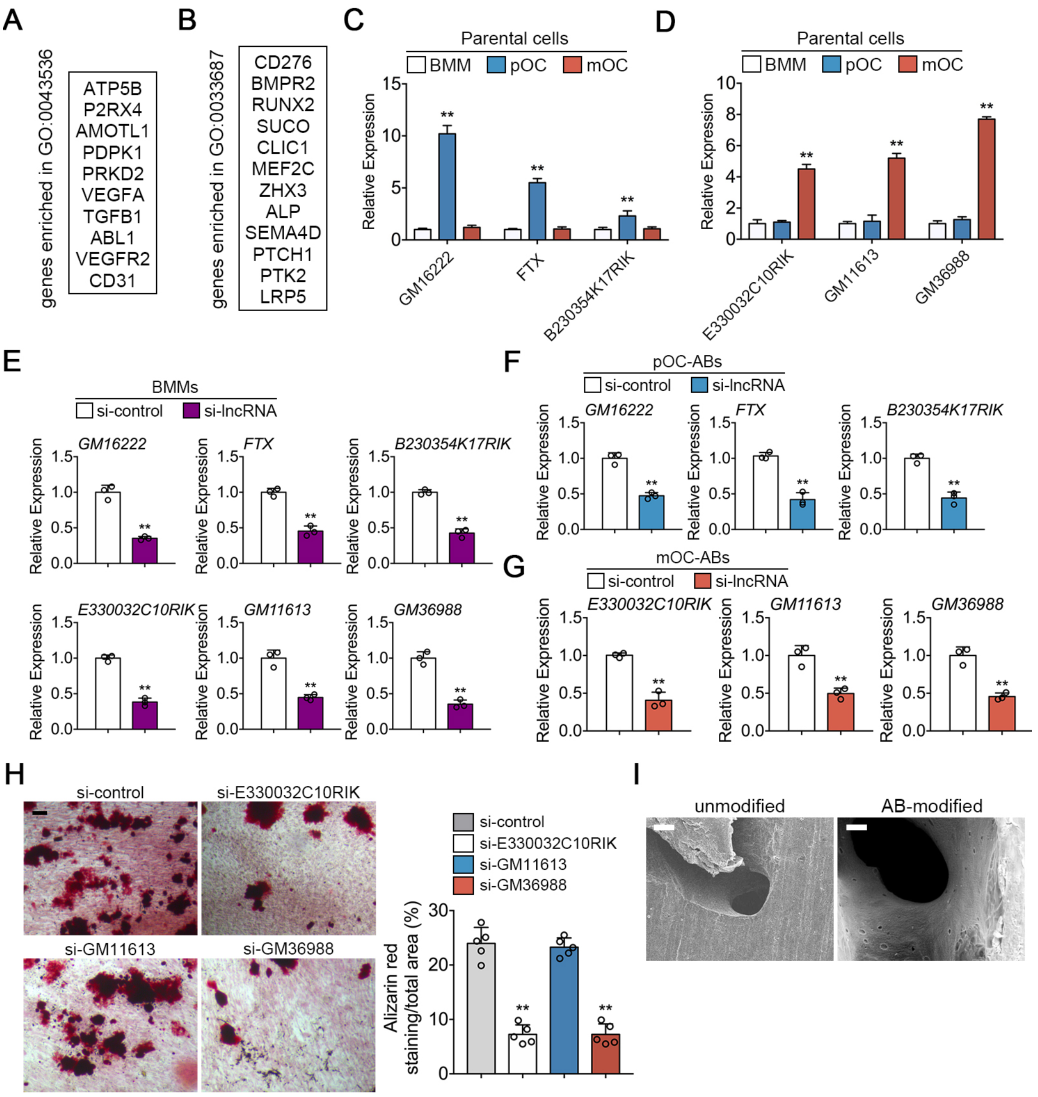

Supplementary Figure S6. Functional similarities of ABs with parental osteoclasts are mediated by key lncRNAs

(A) Genes enriched in 'positive regulation of blood vessel endothelial cell migration' (GO:0043536) and (B) 'osteoblast proliferation' (GO:0033687) were listed.

(C) Relative expression levels of lncRNA signatures *GM16222*, *FTX* and *B230354K17RIK* in parental cell pOCs.

(D) Relative expression levels of *E330032C10RIK*, *GM11613* and *GM36988* in parental cell mOCs.

(E) The expression levels of lncRNAs BMMs after transfection with si-lncRNA were measured by qRT-PCR.

(F) The expression levels of *GM16222*, *FTX* and *B230354K17RIK* in pOC-ABs were detected after transfection with respective lentivirus.

(G) The expression levels of *E330032C10RIK*, *GM11613* and *GM36988* in mOC-ABs were detected after transfection with respective lentivirus.

(H) Alizarin red staining results of indicated groups with quantification. Bar represents 100 μm.

(I) Representative scanning electron microscope (SEM) images of unmodified and AB-modified DBM. Bar represents 20 μm.

The data in the figures represent the averages ± SD. Significant differences are indicated as * ($p < 0.05$) or ** ($p < 0.01$) paired using Student's t test unless otherwise specified.

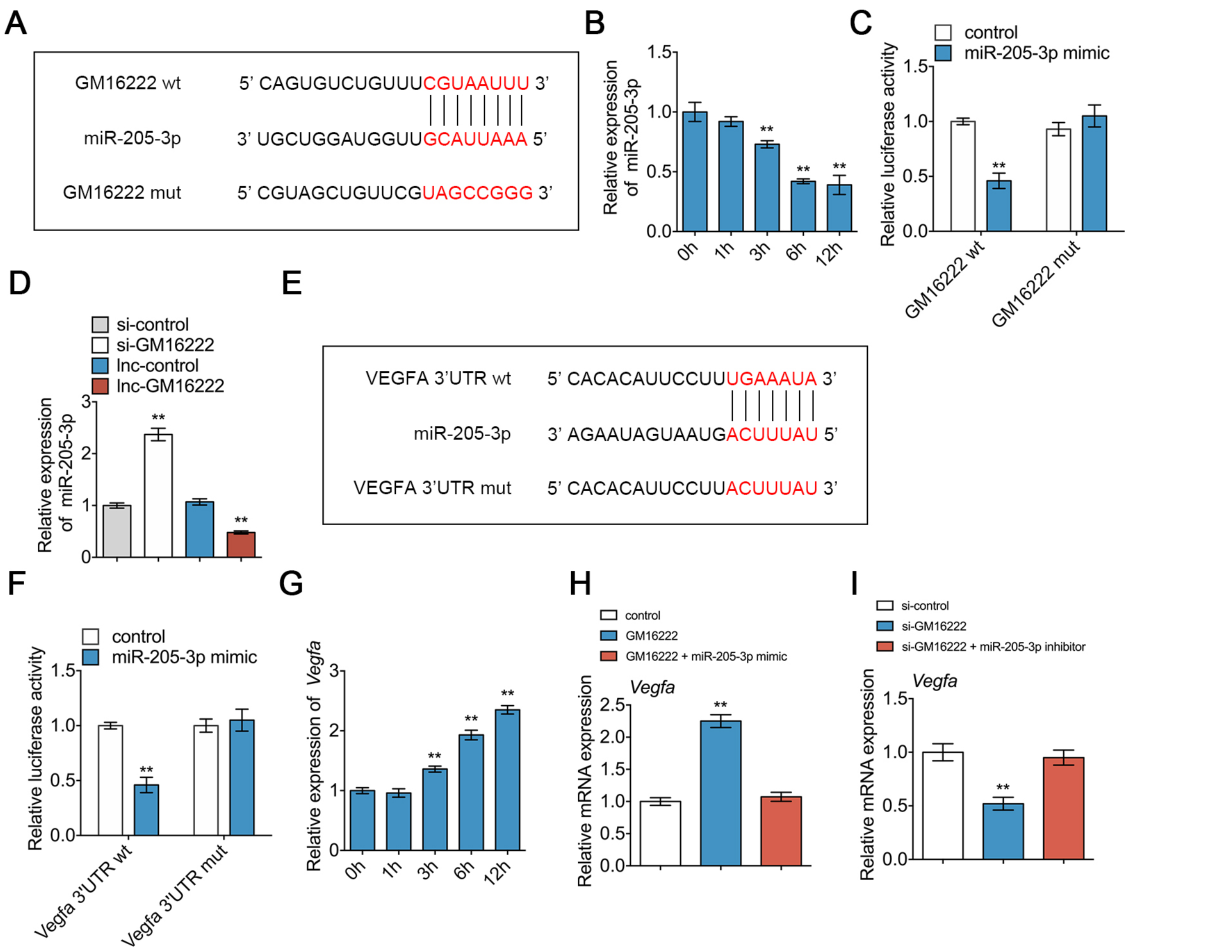

Supplementary Figure 7. pOC-ABs regulates angiogenesis via GM16222 /miR-205-3p/*VEGFA* axis.

(A) Construction of GM16222 3' UTR wild type (wt) and mutant (mut) luciferase reporter vectors.

(B) Relative expression levels of miR-205-3p during tube formation of EPCs.

(C) Relative luciferase activity of reporter containing the 3' UTR of GM16222 upon cotransfection with miR-205-3p, relative to transfection with negative control miRNA, n = 5.

(D) Relative expression levels of miR-205-3p in EPCs knockdown or overexpressed GM16222, n = 3.

(E) Construction of *VEGFA* 3' UTR wild type (wt) and mutant (mut) luciferase reporter vectors.

(F) Relative luciferase activity of reporter containing the 3' UTR of *VEGFA* upon cotransfection with miR-205-3p, relative to transfection with negative control miRNA, n = 5.

(G) Relative expression levels of *VEGFA* during tube formation of EPCs.

(H) Relative expression levels of *VEGFA* in EPCs transfected with GM16222, GM16222 and miR-205-3p mimics.

(I) Relative expression levels of *VEGFA* in EPCs transfected with si-GM16222, si-GM16222 and miR-205-3p inhibitor.

The data in the figures represent the averages ± SD. Significant differences are indicated as * ($p < 0.05$) or ** ($p < 0.01$) paired using Student's t test unless otherwise specified.

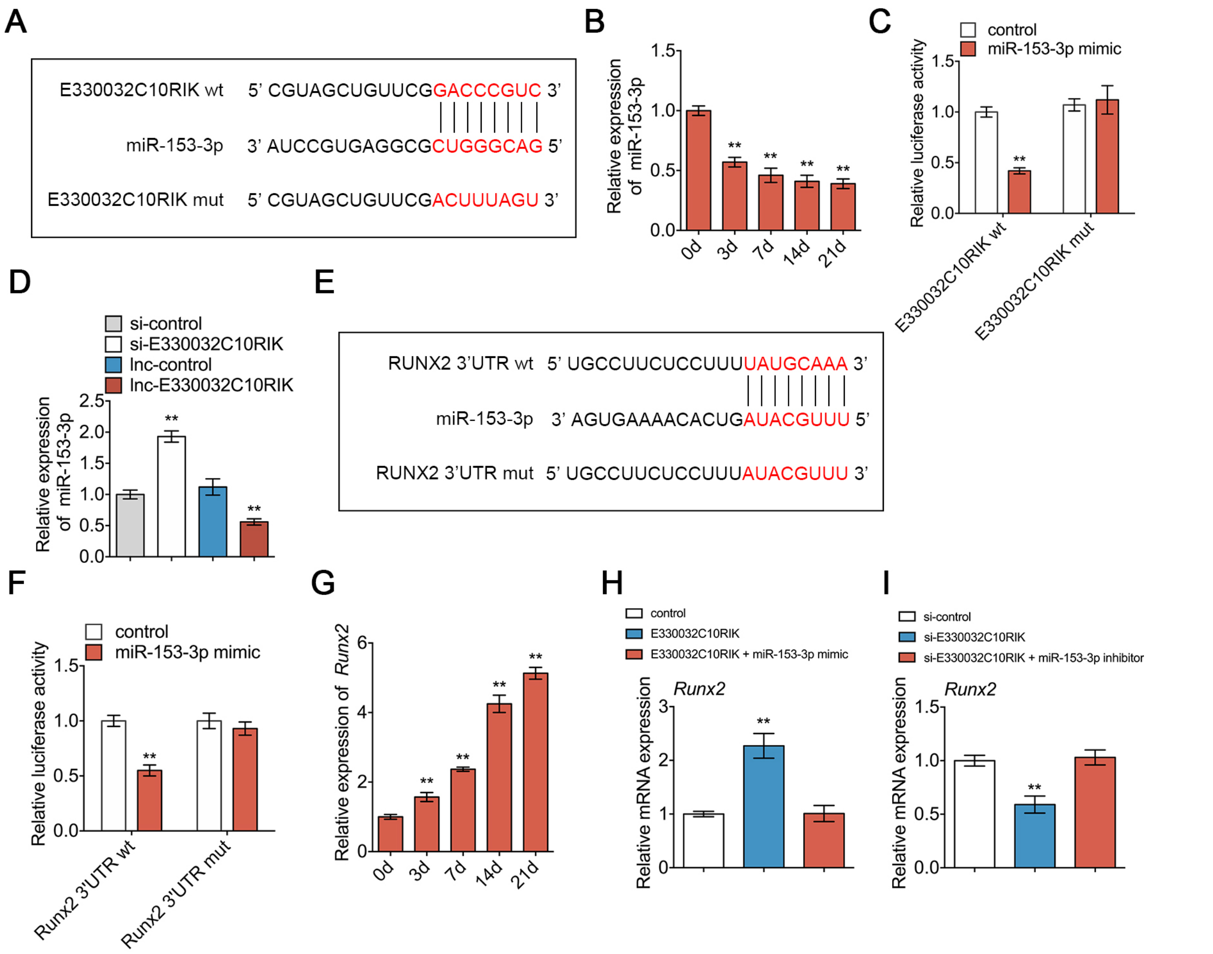

Supplementary Figure 8. mOC-ABs promotes osteogenic differentiation through E330032C10RIK/miR-153-3p/*RUNX2* axis.

(A) Construction of E330032C10RIK 3' UTR wild type (wt) and mutant (mut) luciferase reporter vectors.

(B) Relative expression levels of miR-153-3p during osteogenic differentiation of MC3T3-E1 cells.

(C) Relative luciferase activity of reporter containing the 3' UTR of E330032C10RIK upon cotransfection with miR-153-3p, relative to transfection with negative control miRNA, n = 5.

(D) Relative expression levels of miR-153-3p in MC3T3-E1 cells knockdown or overexpressed E330032C10RIK, n = 3.

(E) Construction of *RUNX2* 3' UTR wild type (wt) and mutant (mut) luciferase reporter vectors.

(F) Relative luciferase activity of reporter containing the 3' UTR of *RUNX2* upon cotransfection with miR-153-3p, relative to transfection with negative control miRNA, n = 5.

(G) Relative expression levels of *RUNX2* during osteogenic differentiation of MC3T3-E1 cells.

(H) Relative expression levels of *RUNX2* in MC3T3-E1 cells transfected with E330032C10RIK, E330032C10RIK and miR-153-3p mimics.

(I) Relative expression levels of *RUNX2* in MC3T3-E1 cells transfected with si-E330032C10RIK, si-E330032C10RIK and miR-153-3p inhibitor.

The data in the figures represent the averages ± SD. Significant differences are indicated as * ($p < 0.05$) or ** ($p < 0.01$) paired using Student's t test unless otherwise specified.

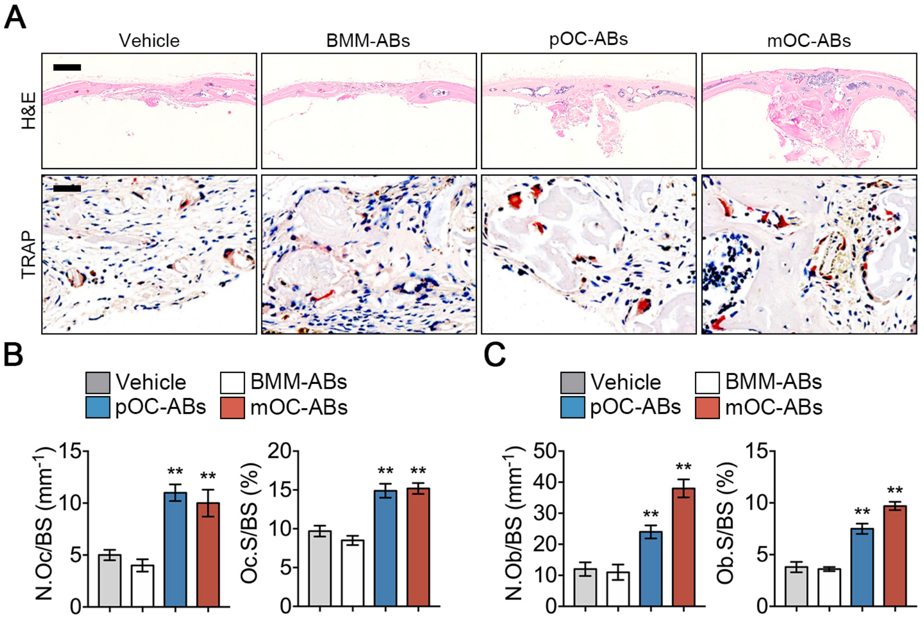

Supplementary Figure 9. mOC-ABs promote osteogenic differentiation *in vivo*

(A) Representative H&E and TRAP staining images of mice grafted with AB-DBM for 4 weeks. Bar represent 1 mm in H&E staining and 50 μm in TRAP staining.

(B) Quantitative analysis of the number of osteoclasts (N.Oc) on cortical bone surface (BS) and osteoclast surface per bone surface (Oc.S/BS) using TRAP staining in indicated groups, n = 8 per group.

(C) Quantitative analysis of the number of osteoblasts (N.Ob) on cortical bone surface (BS) and osteoblasts surface per bone surface (Ob.S/BS) using OCN immunohistochemical staining in indicated groups, n = 8 per group.

The data in the figures represent the averages ± SD. Significant differences are indicated as * ($p < 0.05$) or ** ($p < 0.01$) paired using Student's t test unless otherwise specified.

Supplementary Table S1. Primer sequences for qPCR

Genes	Forward	Reverse	Tm (°
Runx2	5'-ATGCTTCATTCGCCTCACAAA-3'	5'-GCACTCACTGACTCGGTTGG-3'	61
Alpl	5'-AACCCAGACACAAGCATTCC-3'	5'-GAGACATTTTCCCGTTCACC-3'	60
Pecam1	5'-ACGCTGGTGCTCTATGCAAG-3'	5'-TCAGTTGCTGCCCATTCATCA-3'	62
Kdr	5'-GGCTAACGTGTCCTGCCAG-3'	5'-AGTACCAACGCACAGTGATATTG-3'	62
GM16222	5'-CTTCCTGTAGAGAGCAAGTGT-3'	5'-GTCTTGGATTTTACCTATTGC-3'	60
FTX	5'-TCATGACAAACCTGTTTGT-3'	5'-CTAATCATCTCTGTCCCTA-3'	60
B230354K17RIK	5'-ACTCACTTCCTCCGACTTTAC-3'	5'-ACGTTCACAAGTGGCCTCAC-3'	61
E330032C10RIK	5'-AGCTTGCGTGGTTTTCTTCGGT-3'	5'-AATCTGAATCTGACTTTTGTTG-3'	61
GM11613	5'-CGAAGGAGAGAGAGAGAAAT-3'	5'-CCTTCCCTGGTGAGCTGGATTC-3'	62
GM36988	5'-CGGGATGCAGTTGTAAGTATC-3'	5'-ACTCTCCTACAGTGAGAGGGGT-3'	61
Gapdh	5'- TGGATTTGGACGCATTGGTC-3'	5'- TTTGCACTGGTACGTGTTGAT-3'	62
